# A stem cell roadmap of ribosome heterogeneity reveals a function for RPL10A in mesoderm production

**DOI:** 10.1038/s41467-022-33263-3

**Published:** 2022-09-19

**Authors:** Naomi R. Genuth, Zhen Shi, Koshi Kunimoto, Victoria Hung, Adele F. Xu, Craig H. Kerr, Gerald C. Tiu, Juan A. Oses-Prieto, Rachel E. A. Salomon-Shulman, Jeffrey D. Axelrod, Alma L. Burlingame, Kyle M. Loh, Maria Barna

**Affiliations:** 1 Department of Genetics, Stanford University, Stanford, CA 94305 USA; 2 Department of Biology, Stanford University, Stanford, CA 94305 USA; 3 Department of Pathology, Stanford University, Stanford, CA 94305 USA; 4 Department of Pharmaceutical Chemistry, University of California San Francisco, San Francisco, CA 94158 USA; 5 Department of Developmental Biology, Stanford University, Stanford, CA 94305 USA; 6 Present Address: Genentech Inc, South San Francisco, CA 94080 USA

**Keywords:** Mesoderm, Gene regulation, Ribosome, Stem-cell differentiation

## Abstract

Recent findings suggest that the ribosome itself modulates gene expression. However, whether ribosomes change composition across cell types or control cell fate remains unknown. Here, employing quantitative mass spectrometry during human embryonic stem cell differentiation, we identify dozens of ribosome composition changes underlying cell fate specification. We observe upregulation of RPL10A/uL1-containing ribosomes in the primitive streak followed by progressive decreases during mesoderm differentiation. An *Rpl10a* loss-of-function allele in mice causes striking early mesodermal phenotypes, including posterior trunk truncations, and inhibits paraxial mesoderm production in culture. Ribosome profiling in *Rpl10a* loss-of-function mice reveals decreased translation of mesoderm regulators, including Wnt pathway mRNAs, which are also enriched on RPL10A/uL1-containing ribosomes. We further show that RPL10A/uL1 regulates canonical and non-canonical Wnt signaling during stem cell differentiation and in the developing embryo. These findings reveal unexpected ribosome composition modularity that controls differentiation and development through the specialized translation of key signaling networks.

## Abstract (web summary)

How ribosomes differ in composition and function to regulate gene expression is poorly understood. Here, the authors show that ribosome composition changes during stem cell differentiation and identify a ribosomal protein that regulates production of the mesoderm lineage.

## Introduction

Translation is a crucial regulatory step in the central dogma of gene expression that diversifies and tunes the expression of gene products across cell types and tissues. The extent to which translational control contributes to the regulation of dynamic cellular transitions, such as in stem cell differentiation and embryonic development, remains less characterized. Reliance on specific rates of protein synthesis for proper renewal and differentiation has been reported for multiple stem cell populations^1^, including hematopoietic stem cells, whose functions are inhibited by both increases and decreases in global translation^2^. One hypothesis is that translation may be tailored within each cell type, thereby providing the regulation of gene expression required for proper cell differentiation and organismal development, but the mechanisms underlying this extensive regulation remain poorly understood.

A potential hub for this translational regulation is the ribosome itself, the ancient macromolecular machine, composed of four ribosomal RNAs and 80 core ribosomal proteins (RPs), responsible for protein synthesis across all kingdoms of life^3^. Despite the ubiquity of the ribosome, mutations in different RPs lead to unique tissue-specific phenotypes in animal models^4^ as well as distinct human pathologies, ranging from anemia^5^ to asplenia^6^ to hereditary hair loss^7^, suggesting that individual RPs may have cell type-specific functions. Recently, several RPs have been shown to be substoichiometric in actively translating ribosomes of certain cells, namely HEK293 and mouse embryonic stem cells^8,9^. This heterogeneity in ribosome composition has been suggested to control the translation of distinct subsets of mRNAs^8^. However, whether ribosome composition changes during cellular differentiation and if distinct ribosome types are required to fulfill the unique protein synthesis needs of cells across time and space has yet to be elucidated.

In this work, we address this outstanding question by using relative quantification mass spectrometry to establish a proteomic roadmap of ribosome heterogeneity during stem cell fate specification. We uncover extensive remodeling of actively translating ribosome composition during human embryonic stem cell (hESC) differentiation, including upregulation of the heterogeneous RP RPL10A/uL1 in the primitive streak cell types followed by gradual reduction during paraxial mesoderm differentiation. By creating a unique loss-of-function mouse model, we find that RPL10A/uL1 functions in gastrulation and mesoderm formation and regulates translation of Wnt pathway components, revealing a role for ribosomal proteins in the control of differentiation and development through the selective translation of core developmental signaling networks.

## Results

### Quantitative proteomics of ribosome composition

**Fig. 1:**
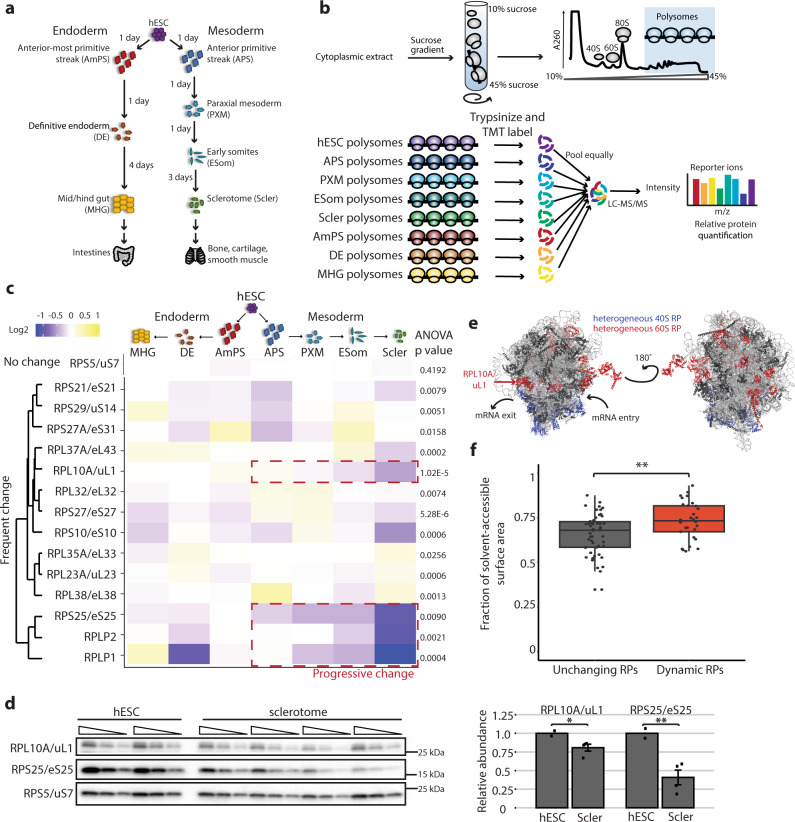
Quantitative proteomics reveals ribosome composition changes during hESC differentiation. **a** Schematic of hESC differentiation down the endoderm lineage to the mid/hindgut and down the mesoderm lineage to the sclerotome. **b** Schematic of polysome isolation by sucrose-gradient fractionation and labeling with tandem mass tags (TMT) to quantify each RP in a differentiated cell sample relative to the hESC starting population. **c** Heatmap of relative polysome abundance for RPs that change significantly by at least 10% in at least two differentiated cell types relative to hESCs. Several show progressive changes in abundance during mesoderm differentiation (red dashed boxes). One RP (RPS5/uS7) that does not change significantly is also included as an illustrative example. Heatmap values are median ratios of polysome abundance in each differentiated cell relative to hESCs in log2 scale, *n* = 6 for each cell type except for anterior primitive streak and mid/hindgut (*n* = 7 each), and *P* values for each RP were calculated by ANOVA. **d** Western blot of hESC (*n* = 2) and sclerotome (*n* = 4) polysome samples, each loaded as a serial dilution (1, 0.5, 0.25 µg). RPL10A/uL1 and RPS25/eS25 expression were normalized to the non-heterogeneous RPS5/uS7 and the middle dilution (0.5 µg) was used for quantification (shown as mean +/− SEM). Student’s *t* test *P* value = 0.03 for RPL10A/uL1, 0.008 for RPS25/eS25. **e** Location of the 31 heterogeneous RPs in the polysome mass spectrometry on the human 80 S ribosome (PDB: 4v6x). Small and large subunit heterogeneous RPs are blue and red, respectively. Non-heterogeneous RPs are dark gray and rRNA light gray. RPL10A/uL1 (red arrow) is located near the mRNA exit tunnel. **f** Fraction of the surface area that is solvent-exposed for each RP on the human ribosome. The 31 RPs that change significantly in polysomal abundance are significantly more solvent-accessible (Mann–Whitney test *P* = 0.0055), indicating they are enriched at the surface of the ribosome. Box is the interquartile range (IQR), center line is the median, whiskers represent 1.5*IQR from the box boundaries, and each point represents a single RP. *P value < 0.05; **P value < 0.01. Source data are provided as a Source Data file.

To define the changes in ribosome composition during early developmental stages, we took advantage of recent innovations in stem cell differentiation to direct H7 human embryonic stem cells (hESCs) down endoderm and mesoderm lineages, representing some of the earliest cell fate decisions made by a developing embryo^10,11^ (Fig. 1a). For the endoderm lineage, we progressively differentiated hESCs into an anterior-most primitive streak, then definitive endoderm, and finally into mid/hindgut, the progenitor of the intestines; for the mesoderm lineage, we differentiated hESCs into anterior primitive streak followed by paraxial mesoderm, then early somites, and culminating in sclerotome, the precursor of the bone and cartilage of the axial skeleton. Cell fates were confirmed by qPCR analysis of known genetic markers (Supplementary Fig. 1). In this way, we were able to rapidly induce differentiation at the scales and purities required for quantitative analysis down two of the three embryonic germ layers—representing a wide array of cell types present at different stages of embryonic development—and to identify changes in ribosome composition in as short a window as 24 h of differentiation.

To identify changes in ribosome composition that are most likely to directly impact the translation capacity of the cell, we first focused on actively translating ribosomes (polysomes). From each differentiated cell population (anterior primitive streak, paraxial mesoderm, early somites, sclerotome; anterior-most primitive streak, definitive endoderm, mid/hindgut) and undifferentiated hESCs, we collected cytoplasmic extracts and performed sucrose-gradient density ultracentrifugation to separate ribosomal species (free subunits, 80S monosomes, and polysomes) into different fractions (Fig. 1b and Supplementary Fig. 2). We pooled together the fractions corresponding to three or more ribosomes to isolate polysomal RPs, which correspond to mature, actively translating ribosomes. To quantify changes in the abundance of each core RP, we used tandem mass tag (TMT) labeling to add a unique chemical tag to the peptides from each cell type^12^. This allowed us to quantify the relative RP abundance in each differentiated cell type relative to an undifferentiated hESC control sample by measuring the ratio of the two TMT labels. We quantified 77 out of the 80 core RPs and 1 RP paralog (RPL22L/eL22L), with on average eight peptides used to identify each protein (Supplementary Data 1); other paralogs and three RPs with few tryptic peptides (RPL39/eL39, RPL40/eL40, RPL41/eL41) were not included in the analysis.

In each differentiated cell type, the majority of RPs remain unchanged. However, a subset of the RPs is heterogeneous, showing either increased or decreased polysomal abundance with respect to the differentiation status of the cell (Supplementary Fig. 3 and Supplementary Data 1). In more terminally differentiated cell types, decreases in specific RP incorporation into polysomes occur more frequently and with greater magnitude, suggesting a gradual refinement of ribosome compositions to a more restricted selection as a consequence of cellular differentiation (Supplementary Fig. 3). In all, 31 RPs change significantly in polysome abundance over the course of cellular differentiation (*P* < 0.05 by ANOVA using relative polysomal abundances from all biological replicates) (Supplementary Fig. 3 and Supplementary Data 1), including those previously identified as substoichiometric in actively translating ribosomes in mouse embryonic stem cells^8^ (RPL10A/uL1, RPL38/eL38, RPL11/uL5, RPS25/eS25, RPS7/eS7) and in HEK293 ribosomes^9^ (RPS25/eS25, RPS10/eS10), suggesting that their stoichiometry is dynamically regulated. While several of these heterogeneous RPs show only modest changes in a subset of the differentiated cell types, on the contrary, 14 change by at least 10% in polysomal abundance in at least two differentiated cell types relative to hESCs, with some showing as much as a 50% change (Fig. 1c). This is significant as there are millions of ribosomes in a cell and therefore even a 10% difference would result in hundreds of thousands of ribosomes with a unique composition. Strikingly, several RPs show progressive changes in polysomal abundance as the cells proceed down a lineage: for instance, RPL10A/uL1 is initially upregulated in the primitive streak polysomes, but shows a progressive decrease in abundance through the paraxial mesoderm, early somites, and sclerotome (Fig. 1c, boxed). Many of these progressive changes along the mesoderm lineage are in fact statistically significant by linear regression, including RPL10A/uL1 (*P* = 0.0007, Supplementary Data 1). We further measured the abundance of RPs with good commercially available antibodies such as RPL10A/uL1 as well as RPS25/eS25 in hESC and sclerotome polysomes by western blot and observed similar decreases in abundance as those measured by mass spectrometry (Fig. 1d). Similarly, we also confirmed the increased abundance of RPL23A/uL23 in sclerotome polysomes relative to hESC polysomes by western blot (Supplementary Fig. 4A). These findings not only suggest that actively translating ribosomes are highly dynamic in their composition, but also that RP abundance in the polysomes may be attuned to the differentiation state of the cell. The 31 heterogeneous RPs have statistically significantly increased solvent accessibility compared to non-heterogeneous RPs, indicating that they are enriched on the surface of the ribosome, and include RPs positioned near the mRNA entry and exit channels^13^ (Fig. 1e, f and Supplementary Movie 1), suggesting that these RPs could specialize the ribosome for translation of transcripts via contacts with ribosome-associated proteins and/or direct binding of mRNAs.

### Polysome ribosome composition is regulated at multiple levels

**Fig. 2:**
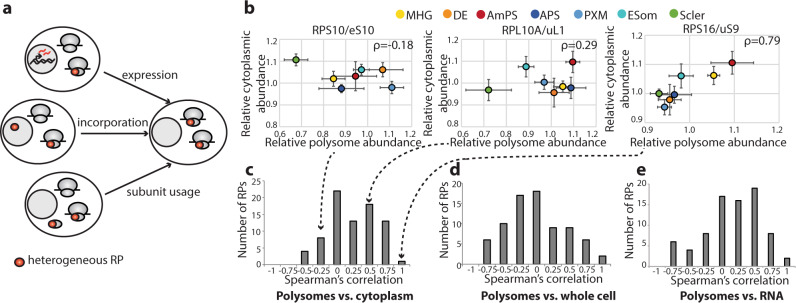
Polysome composition is regulated at multiple levels. **a** Schematic of possible mechanisms regulating changes in RP abundance in the polysomes across cell types. **b** Scatterplots of median polysomal and cytoplasmic protein relative abundance in each differentiated cell relative to hESCs for RPS10/eS10, RPL10A/uL1, and RPS16/uS9 with Spearman’s correlation coefficients (ρ) in upper right corners. Error bars are standard error and points are color-coded by cell type. *N* = 6 for all cell types and sample preparations except for the following: *n* = 7 for anterior primitive streak and mid/hindgut polysome samples and n = 8 for mid/hindgut cytoplasmic samples. Dashed arrows indicate the locations of these RPs in the histogram in (**c**). **c** Histogram of Spearman’s correlation coefficients for each RP comparing polysomal and cytoplasmic protein abundance (same *n* as in (**b**)). **d** Histogram of Spearman’s correlation coefficients for each RP comparing polysomal (same *n* as in (**b**)) and whole-cell protein abundance (*n* = 5 for early somite, paraxial mesoderm, and mid/hindgut whole-cell samples; *n* = 6 for anterior primitive streak, sclerotome, anterior-most primitive streak, and definitive endoderm whole-cell samples). **e** Histogram of Spearman’s correlation coefficients for each RP comparing polysomal (same *n* as in (**b**)) and mRNA abundance (*n* = 3 for mesoderm lineage, *n* = 1 for endoderm lineage). MHG   mid/hindgut, DE   definitive endoderm, AmPS anterior-most primitive streak, APS   anterior primitive streak, PXM   paraxial mesoderm, ESom early somite, Scler sclerotome.

This dynamic regulation of polysome composition during differentiation led us to next ask at what step of polysome formation this heterogeneity arises. The creation of a polysome is a complex process that includes synthesis of the RPs, import of RPs into the nucleolus (where most of the ribosome biogenesis occurs), export of the assembled subunits to the cytoplasm, and finally engagement of these subunits onto mRNA. Regulation of polysome composition across cell types could accordingly occur at multiple points, including (1) modifying RP expression (either at the mRNA level or post-transcriptionally); (2) changing RP incorporation into ribosomal subunits independently from changes in RP expression; or (3) differences in the numbers of ribosome subunits containing a given RP that are actively engaged in translation (Fig. 2a). To identify which of these mechanisms govern RP abundance in the polysomes, we accordingly expanded our TMT mass spectrometry screen to also include cytoplasmic extract and whole-cell lysates. We further supplemented the protein expression data with reanalysis of existing RNA-seq data^10,11^ for these cell types to measure the mRNA expression level of each RP across cell differentiation. When we compared the polysomal and cytoplasmic protein abundance for each RP across different cell types, we noted a wide range of correlation values: for instance, RPS16/uS9 showed a strong correlation (Spearman’s correlation coefficient ( *ρ*) = 0.79), while RPL10A/uL1 had only mediocre correlation ( *ρ* = 0.29), and RPS10/eS10 was in fact modestly anti-correlated ( *ρ* = −0.18) (Fig. 2b, c and Supplementary Fig. 5). A wide range of correlation values were similarly obtained when comparing polysome to the whole-cell abundance or mRNA abundance (Fig. 2D, E). We observed an even greater number of RPs showing little to no correlation between cytoplasmic extract and whole-cell protein extracts (Supplementary Figs. 4B and 5), which may be explained by differences in composition between cytoplasmic ribosomes and nuclear ribosomal intermediates. Similarly, the correlation between mRNA levels and whole-cell protein expression was also frequently poor (Supplementary Figs. 4B and 5), suggesting extensive posttranscriptional regulation of RP expression, which is in keeping with recent observations of poor concordance between RP mRNA and protein levels in human tissues^14^. The fact that different RPs show distinct patterns of correlation, with some showing positive correlation across all datasets while others show discordance between sample types (Supplementary Fig. 5), suggests that multiple mechanisms are differentially utilized to regulate the incorporation of different RPs into actively translating ribosomal subunits.

The lack of correlation between cytoplasmic and polysomal abundance was particularly surprising, as it raises the intriguing possibility that ribosome composition may differ between actively translating and inactive ribosome populations. For further confirmation of RPs whose incorporation into ribosomes differ between actively translating and inactive ribosome populations, we focused on RPL10A/uL1 and RPS25/eS25, which both showed dynamic changes in polysome abundance during cellular differentiation without changing significantly at the whole cell or cytoplasmic levels (Supplementary Data 1 and Supplementary Fig. 4C). As RPL10A/uL1 and RPS25/eS25 are most strongly downregulated in the polysomes of sclerotome, we performed TMT mass spectrometry on sclerotome and hESC free ribosomal subunits as well as monosomes/disomes and compared RPL10A/uL1 and RPS25/eS25 abundance in these fractions to the polysomal, cytoplasmic, and whole-cell extract data. Supporting this notion, we found that both RPs showed increased abundance in the sclerotome free subunits and monosomes/disomes relative to the polysomes (Supplementary Fig. 4D), in agreement with a model where the polysomal abundance of these RPs is regulated by subunit engagement or elongation on mRNAs: both the sclerotome and hESCs have similar amounts of RPL10A/uL1- and RPS25/eS25-containing ribosomal subunits, but more of these subunits are engaged in mRNA translation in hESCs compared to sclerotome. Therefore, control of ribosome heterogeneity may occur at multiple levels, and these findings suggest that ribosome composition may be modified by cytoplasmic remodeling or regulation of RP composition within polysomes.

### RPL10A/uL1 is required for paraxial mesoderm formation

What is the purpose of these lineage-specific changes in ribosome composition? One possibility is that these reflect differential requirements for individual RPs in specific cell types. In particular, we hypothesized that heterogeneous RPs may have important specialized translation functions in the cell types where they are highest in polysomal abundance. To see if this is the case, we sought a method to selectively regulate the activities of individual RPs on the ribosome in vivo. While classical genetic methods, such as gene deletions and knockdowns, have been employed for multiple RPs, these large-scale perturbations are suboptimal due to the multifaceted functions of RPs both on assembled cytoplasmic ribosomes and during ribosome biogenesis within the nucleolus^15^. Accordingly, decreased expression of certain RPs can lead to defects in ribosome assembly, nucleolar stress and decreased global protein synthesis^5,16–18^, whose effects in turn may mask any phenotypes resulting from loss of cytoplasmic functions on mature ribosomes. We, therefore, sought to uncouple the nucleolar housekeeping roles from cytoplasmic translation activities by creating targeted loss-of-function mutations where the RP is still expressed and incorporated into the ribosome.

**Fig. 3:**
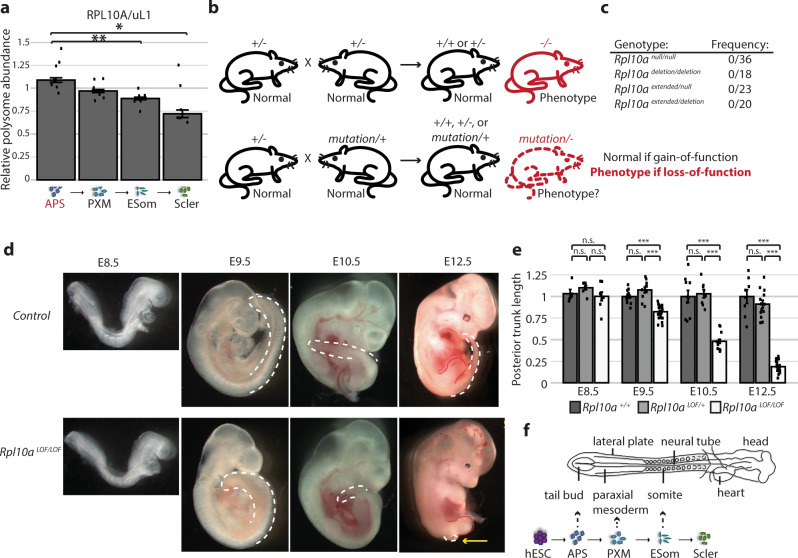
A loss-of-function mouse model uncovers a specialized role for RPL10A/uL1 in the paraxial mesoderm lineage. **a** Median relative abundance of RPL10A/uL1 in the polysomes of the mesoderm lineage relative to hESCs. *N* = 6 for all cell types, except *n* = 7 for primitive streak and error bars are standard error. Student’s *t* test *P* values: anterior primitive streak to early somites 0.006, anterior primitive streak to sclerotome 0.03. **b** Schematic of the genetic complementation test to identify loss-of-function mutations. **c** Observed frequencies of *Rpl10a* ^null/null^ homozygotes, *Rpl10a* ^deletion/deletion^ homozygotes, *Rpl10a* ^extended/null^ double heterozygotes, and *Rpl10a* ^extended/deletion^ double heterozygotes. **d** Lateral views of E8.5, E9.5, E10.5, and E12.5 *Rpl10a* ^LOF/LOF^ and control embryos. The tail bud is traced in white and indicates the posterior trunk truncation; the hindlimb is also traced in red on the E12.5 images. **e** Quantification of posterior trunk length at E8.5, E9.5, E10.5, and E12.5. Graph shows average length relative to wild-type with SEM error bars. No significant differences were observed between wild-type and *Rpl10a* ^*LOF/+*^ embryos; *Rpl10a* ^*LOF/LOF*^ embryos were significantly different from both wild-type and *Rpl10a* ^*LOF/+*^ embryos at all stages except E8.5. For E8.5 wild-type *n* = 5, *Rpl10a* ^*LOF/+*^ *n* = 7, *Rpl10a* ^*LOF/LOF*^ *n* = 8; for E9.5 wild-type *n* = 13, *Rpl10a* ^*LOF/+*^ *n* = 11, *Rpl10a* ^*LOF/LOF*^ *n* = 25; for E10.5 wild-type *n* = 10, *Rpl10a* ^*LOF/+*^ *n* = 8, *Rpl10a* ^*LOF/LOF*^ *n* = 15; for E12.5 wild-type *n* = 8, *Rpl10a* ^*LOF/+*^ *n* = 13, *Rpl10a* ^*LOF/LOF*^ *n* = 19. Wild-type vs. *Rpl10a* ^*LOF/LOF*^ Student’s *t* test *P* values: 0.65 (E8.5), 2.55 × 10^−7^ (E9.5), 2.45 × 10^−5^ (E10.5), 1.13 × 10^−5^ (E12.5); *Rpl10a* ^*LOF/+*^ vs. *Rpl10a* ^*LOF/LOF*^ *P* values: 0.10 (E8.5), 5.31 × 10^−6^ (E9.5), 8.89 × 10^−7^ (E10.5), 1.20 × 10^−10^ (E12.5). **f** Schematic of gastrulation at the tail bud to produce the paraxial mesoderm lineage, with the embryonic tissues represented in the in vitro hESC differentiation indicated. APS   anterior primitive streak, PXM   paraxial mesoderm, ESom early somite, Scler sclerotome, *P value < 0.05; **P value < 0.01, ***P value < 0.001. Source data are provided as a Source Data file.

We focused on RPL10A/uL1, a heterogeneous RP that is upregulated in the polysomes of the primitive streak cell types and then progressively declines during differentiation towards the sclerotome (Fig. 3a). We accordingly hypothesized that RPL10A/uL1 may have specialized functions in the cell types where it is most highly abundant in the polysomes and thereby regulate gastrulation and the production of the paraxial mesoderm lineage. We chose to test this model directly at the organismal level using mouse models in order to observe the full complement of embryonic cell types in their native developmental context. We generated a series of *Rpl10a* mouse alleles by using CRISPR/Cas9 with a gRNA targeting the 5’ end of the RPL10A coding sequence to mutate the solvent-exposed N-terminus of RPL10A/uL1, where interactions with target mRNAs and/or protein interactors are most likely to occur. We produced two mouse models with large deletions in the intron between exons 1 and 2 that result in retention of the remaining intronic sequence (Supplementary Fig. 6A). One of these alleles, a deletion of nucleotides 10–102 of the intron, has an in-frame stop codon shortly after the start codon (*Rpl10a* ^null^). However, the second allele, a deletion of nucleotides 2–79 of the intron, results in the insertion of 38 amino acids directly after the initial methionine in an otherwise unchanged RPL10A/uL1 protein sequence, creating an extended RPL10A/uL1 protein that can be observed by Western as a higher molecular weight band (*Rpl10a* ^extended^) (Supplementary Fig. 6A, B). We additionally generated a conditional *Rpl10a* loss-of- function mouse that was crossed with a line ubiquitously expressing Cre recombinase (CMV-Cre) to create an *Rpl10a* allele missing most of its coding sequence (*Rpl10a* ^deletion^) that serves as a traditional deletion knock-out allele (Supplementary Fig. 6D).

The extended, null, and deletion lines showed no phenotypes in heterozygosity, and the *Rpl10a* ^deletion/+^ mice had no significant differences in *Rpl10a* mRNA or protein expression compared to wild-type controls, indicating that there is complete compensation from the wild-type allele (Supplementary Fig. 6E–G). However, complete loss of RPL10A/uL1 is early embryonic lethal, as no *Rpl10a* ^deletion/deletion^ or *Rpl10a* ^null/null^ embryos could be recovered at E9.5 (Fig. 3c). In contrast, *Rpl10a* ^extended/extended^ homozygotes exhibit perinatal lethality, as the homozygotes are observed at the expected Mendelian ratios as late as E18.5 but are not found after birth (Supplementary Fig. 6C). As this *Rpl10a* mutant allele is an extended protein product, we next confirmed that it is in fact a loss-of-function and not a gain-of-function allele. To do so, we crossed the *Rpl10a* ^extended/+^ mice with the *Rpl10a* ^null/+^ or *Rpl10a* ^deletion/+^ lines to perform the classic, well-established genetic complementation experiment often used to categorize uncharacterized mutations, such as those created in forward genetic screens^19–21^ (Fig. 3b). If the extended *Rpl10a* allele were a gain-of-functionmutation, it would rescue the *Rpl10a* ^null/null^ and *Rpl10a* ^deletion/deletion^ phenotype of early embryonic lethality; however, if it were a loss-of-function mutation, it would fail to complement, and the *Rpl10a* ^*extended/deletion*^ and *Rpl10a* ^*extended/null*^ compound heterozygote embryos would not be viable. We did not recover any compound heterozygote (*Rpl10a* ^extended/null^ or *Rpl10a* ^extended/deletion^) progeny postnatally or at mid-gestation from these crosses (Fig. 3c), indicating that the extended *Rpl10a* allele cannot complement the loss of RPL10A/uL1 and thus is a hypomorphic loss-of-function mutation. The extended allele will accordingly be referred to as a loss-of-function (*Rpl10a* ^LOF^) from here on.

*Rpl10a* ^LOF/LOF^ homozygous mutant mice exhibit a unique array of tissue-specific phenotypes including brain abnormalities, edema, ectopic blood vessels, and most prominently a truncation of the trunk, where the tissue posterior to the hindlimbs is absent, which occurs with complete penetrance (Fig. 3d, e). This axial shortening begins at E9.5 (Fig. 3e) and suggests a defect in gastrulation, a process that begins the formation of the three germ layers at the primitive streak—where notably RPL10A/uL1 is upregulated in the polysomes—and continues to ensue at the tail bud to produce the paraxial mesoderm needed for elongation of the embryo (Fig. 3f). While this truncation phenotype has to our knowledge never been previously reported for any RP mouse model, we further sought to confirm the specificity of this phenotype for RPL10A/uL1 by also employing a conditional allele for RPS6/eS6. RPS6/eS6 is an essential ribosomal protein with critical functions in ribosome biogenesis and is not significantly altered in polysome abundance during hESC differentiation. The *Rps6* conditional allele is a gold-standard model for ribosome dysfunction with one of the most severe phenotypes, as even loss of one copy of *Rps6* results in early embryonic lethality prior to gastrulation, and tissue-specific *Rps6* haploinsufficiency models have been employed across multiple organs and developmental stages to study the outcomes of ribosomal perturbation^22–26^. To this end, we employed a Brachyury Cre transgenic mouse (*T*^*Cre*^)^27^ to inactive RPS6. Brachyury is active in progenitor cells that reside within the primitive streak and tail bud and which give rise to lineages emerging from these tissues as the embryonic axis extends. *T* ^Cre^; *Rps6* ^lox/+^ mice do not have posterior trunk truncations (Supplementary Fig. 7), indicating that this phenotype is not due to global ribosomal perturbations but rather to the loss of a unique RPL10A/uL1 function in the mesoderm.

**Fig. 4:**
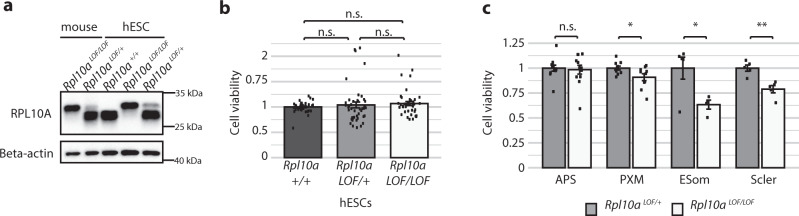
*Rpl10a* loss-of-function hESCs have defects in paraxial mesoderm production. **a** Western of whole-cell lysates from *Rpl10a* ^LOF/LOF^ and *Rpl10a* ^LOF/+^ embryos, unedited hESCs, and hESC clones either homozygous or heterozygous for the *Rpl10a* LOF insertion. **b** Cell viability of wild-type, heterozygous, and homozygous *Rpl10a* ^LOF/LOF^ hESCs as measured by Cell Titer Glo (*n* = 44). Data are shown as mean +/− SEM and significance was calculated using Student’s *t* tests. **c** Cell viability of heterozygous and homozygous *Rpl10a* ^LOF/LOF^ hESCs as measured by Cell Titer Glo during differentiation down the paraxial mesoderm lineage (*n* = 12 for each cell line for anterior primitive streak, *n* = 10 for each cell line for paraxial mesoderm, *n* = 5 for each cell line for sclerotome, *n* = 5 for *Rpl10a* ^*LOF/+*^ cells and *n* = 4 for *Rpl10a* ^*LOF/LOF*^ cells for early somites). Data are shown as mean +/− SEM. Student’s *t* test *P* values: 0.77 (anterior primitive streak), 0.04 (paraxial mesoderm), 0.03 (early somite), 0.002 (sclerotome). APS   anterior primitive streak, PXM   paraxial mesoderm, ESom early somite, Scler sclerotome. *P value <0.05; **P value <0.01. Source data are provided as a Source Data file.

To determine whether the *Rpl10a* loss-of-function allele would also perturb mesoderm formation in our in vitro stem cell differentiation system, we used CRISPR/Cas9 to endogenously edit the *Rpl10a* locus in hESCs (Supplementary Fig. 8A). We inserted the sequence for the 38 amino acid N-terminal extension directly after the start codon, resulting in an identical protein product to the mouse loss-of-function allele (Fig. 4a). Wild-type, heterozygous, and homozygous *Rpl10a* ^*LOF/LOF*^ hESCs did not differ in their viability (Fig. 4b). When differentiated down the paraxial mesoderm lineage, *Rpl10a* ^*LOF/LOF*^ cells were capable of inducing expression of the expected lineage markers, though interestingly several showed altered expression relative to controls, including decreased expression of *Uncx4.1* in the sclerotome (Supplementary Fig. 8B). Most importantly, however, *Rpl10a* ^*LOF/LOF*^ cells exhibited significantly reduced cell viability upon induction of the paraxial mesoderm fate, with further decreases in viability as the cells proceeded down the lineage to the early somites and sclerotome (Fig. 4c). This indicates that the loss-of-function mutation in *Rpl10a* compromises the production of the paraxial mesoderm lineage in our in vitro differentiation model, paralleling the phenotype observed in the mouse.

**Fig. 5:**
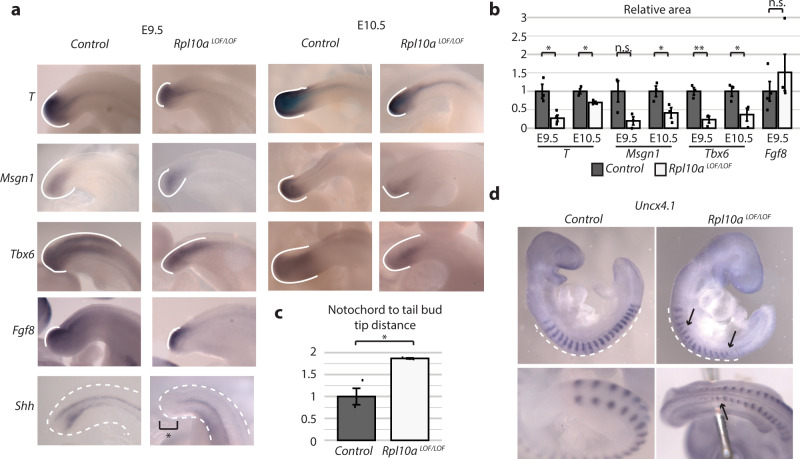
*Rpl10a*^*LOF/LOF*^ embryos exhibit defects in paraxial mesoderm lineage formation. **a** Whole-mount in situ hybridizations in control and *Rpl10a* ^LOF/LOF^ embryos at E9.5 and E10.5. The region of expression at the tail bud is demarcated with a solid white line. A dashed white line outlines the tail bud in the *Shh* in situ. **b** Quantification of the area of staining relative to total tail bud area. For each probe, stage, and genotype *n* = 3 with the exception of *Fgf8* (*n* = 4 for each genotype) and *T* (*n* = 4 for E9.5 *Rpl10a* ^*LOF/LOF*^). Data are shown as mean +/− SEM, Student’s *t* test *P* values: 0.04 (*T* E9.5), 0.03 (*T* E10.5), 0.09 (*Msgn1* E9.5), 0.045 (*Msgn1* E10.5), 0.005 (*Tbx6* E9.5), 0.049 (*Tbx6* E10.5), 0.40 (*Fgf8* E9.5). **c** Quantification of the distance between the end of the notochord, as demarcated by *Shh* in situ, and the tip of the tail bud (*n* = 3). Distances were normalized to the distance between limb buds and otic placode in each embryo to account for any differences in embryo size. Data are shown as mean +/− SEM, and significance was calculated using Student’s *t* tests (*P* value = 0.04). **d** Whole-mount in situ hybridizations for *Uncx4.1* in control and *Rpl10a* ^LOF/LOF^ embryos at E9.5, lateral view (top), and view of the posterior end of the embryo (bottom). The dashed white line demarcates the region of expression; black arrows indicate abnormalities in somite boundaries and spacing. *P value < 0.05; **P value < 0.01. Source data are provided as a Source Data file.

To further characterize the posterior truncation phenotype, we performed whole-mount in situ hybridizations for mesodermal markers on E9.5 and E10.5 *Rpl10a* ^LOF/LOF^ and control embryos (Fig. 5). *Brachyury* (*T*, essential for mesoderm production), *Msgn1* (required for presomitic mesoderm differentiation), and *Tbx6* (required for paraxial mesoderm formation), show restricted expression at the tail bud of E9.5 and E10.5 *Rpl10a* ^LOF/LOF^ embryos (Fig. 5a, b). *Rpl10a* ^LOF/LOF^ embryos also exhibit a shortened notochord (as labeled by Sonic Hedgehog (*Shh*) expression), an additional consequence of defects in axis elongation (Fig. 5a, c). However, *Fgf8* expression is unperturbed in *Rpl10a* ^LOF/LOF^ embryos, indicating that the tail bud structure and mesoderm signaling pathways are not universally disrupted (Fig. 5a, b). We additionally did not observe perturbations in the expression of the neural progenitor marker *Sox2* in *Rpl10a* ^LOF/LOF^ embryos (Supplementary Fig. 9A). Probing for *Uncx4.1* revealed defects in somitogenesis, as somites were irregularly spaced and had indistinct boundaries, particularly at the posterior end of the embryo (Fig. 5d). Indeed, the improper patterning and fusion of these segments was even more apparent upon cartilage staining of E14.5 embryos, which revealed vertebral fusions and lack of well-defined posterior vertebral elements (Supplementary Fig. 9B). By E17.5, *Rpl10a* ^LOF/LOF^ embryos exhibit profound axial skeleton defects: bone and cartilage staining revealed little separation of vertebral segments and almost no ossification, particularly at the posterior end, as well as fragments of cartilage unattached to either the spine or sternum instead of a fully enclosed rib cage (Supplementary Fig. 9C). Remarkably, the development of skeletal elements derived from embryonic sources other than the paraxial mesoderm showed little disruption: the skull was grossly normal, as were the limbs, with the exception of a mildly decreased ossification in the radius and the completely penetrant loss of the posterior digit in all four limbs (Supplementary Fig. 9C).

### *Rpl10a*^LOF/LOF^ embryos have reduced translation of Wnt pathway genes

**Fig. 6:**
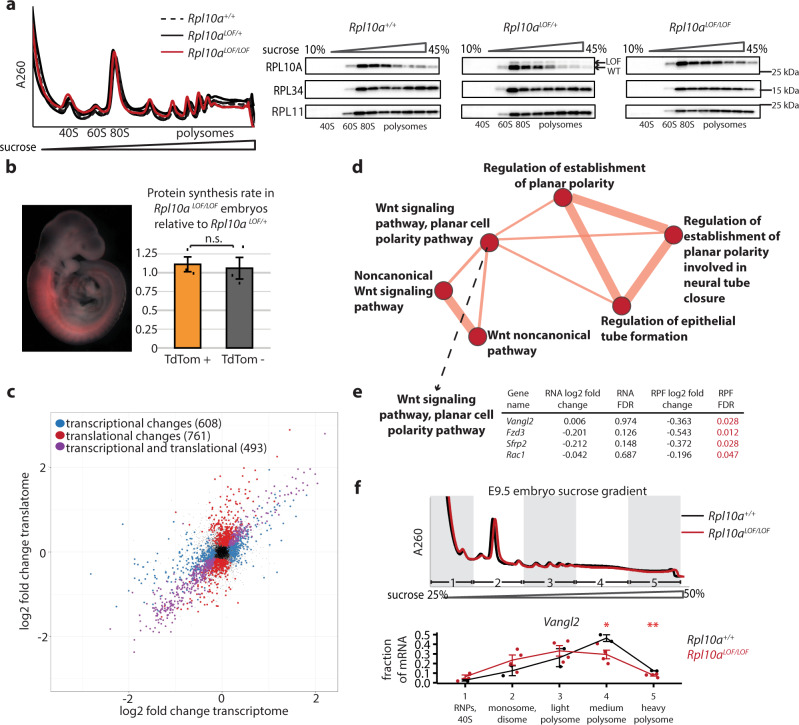
*Rpl10a*^*LOF/LOF*^ embryos exhibit reduced translation of Wnt signaling components. **a** (Left) Polysome traces of wild-type, *Rpl10a* ^LOF/+^, and *Rpl10a* ^LOF/LOF^ whole E13.5 embryo extracts show similar distributions of polysomes, monosomes, and free subunits. (Right) Western blots of protein precipitated from each sucrose-gradient fraction show the extended RPL10A/uL1 protein is incorporated into polysomes equivalently to wild-type. **b** (Left) Fluorescence image of E9.5 *Meox1* ^Cre^; *Ai9* embryo illustrating the presomitic/somitic mesoderm tdTomato reporter expression. (Right) OP-Puromycin incorporation rates in *Meox1* ^Cre^; Ai9; *Rpl10a* ^LOF/LOF^ E9.5 presomitic/somitic mesoderm (tdTom+) and in the rest of the embryo (tdTom−) (*n* = 3). Values are normalized to OP-Puro incorporation rates of TdTom+ and TdTom– cells of *Meox1* ^Cre^; Ai9; *Rpl10a* ^LOF/+^ E9.5 control littermates. Data shown are mean +/− SEM, and significance was calculated using Student’s *t* test. **c** Comparison of RNA-seq and ribosome profiling for whole E8.5 *Rpl10a* ^LOF/LOF^ and wild-type embryos (*n* = 3 each). Genes changing significantly (false discovery rate (FDR) < 0.1) only in mRNA abundance are blue; genes changing significantly (FDR < 0.1) only in ribosome occupancy are red; genes changing significantly in both datasets are purple. **d** Selected Wnt signaling gene sets that were significantly (FDR < 0.1) enriched in CAMERA gene set enrichment analysis of genes with altered translation efficiency in *Rpl10a* ^LOF/LOF^ embryos compared to wild-type. All gene sets shown are translationally downregulated. Node size = gene set size; edge size = gene set overlap. **e** Genes from the “Wnt signaling pathway, planar cell polarity pathway” set with statistically significant changes in ribosome-protected footprints (RPF) but not in total RNA-Seq (RNA). **f** E9.5 wild-type (*n* = 2) and *Rpl10a* ^*LOF*/LOF^ (*n* = 4) gradient RT-qPCR for *Vangl2* showing the fraction of the total mRNA found in each of the five fractions of the 25–50% sucrose gradient demarcated in the schematic. Data shown are mean +/− SEM. *Vangl2* is significantly decreased in *Rpl10a* ^*LOF*/LOF^ medium and heavy polysomes relative to wild-type (Student’s *t* test *P* values = 0.04 and 0.008, respectively). *P value < 0.05; **P value < 0.01. Source data are provided as a Source Data file.

The specificity of these phenotypes and their correspondence with the cell types where RPL10A/uL1 is heterogeneous in the polysomes suggest that RPL10A/uL1 may play a highly important role in the regulation of the paraxial mesoderm lineage, and so we next sought to determine molecularly how this occurs. We first examined whether the pronounced *Rpl10a* ^LOF/LOF^ developmental phenotypes are accompanied by changes in global protein synthesis. Sucrose-gradient fractionation from wild-type, heterozygous, and *Rpl10a* ^LOF/LOF^ mutant embryos had similar polysome profiles (Fig. 6a). Identical polysome profiles were also obtained from wild-type, heterozygous, and *Rpl10a* ^*LOF/LOF*^ hESCs (Supplementary Fig. 10A). Western blots of protein precipitated from each embryo sucrose gradient fraction additionally revealed the *Rpl10a* ^LOF^ allele is normally incorporated into ribosomes, including the polysomes, similarly to the wild-type. To directly compare protein synthesis rates, we measured O-propargyl-puromycin (OPP) incorporation into nascent polypeptide chains in wild-type, heterozygous, and homozygous mutant E9.5 embryos, as well as in wild-type, heterozygous, and homozygous mutant hESCs, and saw no significant differences between the genotypes (Supplementary Fig. 10B, C). As the effects on protein synthesis could be restricted to the paraxial mesoderm lineage where the mouse phenotypes manifest, we additionally measured OPP incorporation specifically in this tissue. We generated *Rpl10a* ^LOF/LOF^ mice with a conditional tdTomato reporter (Ai9), and a Cre recombinase knocked into the *Meox1* locus, which is activated in the presomitic mesoderm, thereby labeling the presomitic mesoderm and its derivatives with tdTomato^28^ (Fig. 6b). We measured OPP incorporation in both the tdTomato-positive cells (presomitic mesoderm and somites) and tdTomato-negative cells (all other tissues) in these embryos at E9.5 and did not see significant differences between *Rpl10a* ^LOF/LOF^ embryos and controls (Fig. 6b), suggesting that the *Rpl10a* ^LOF/LOF^ phenotypes are not driven by global changes in translation.

To molecularly define the function of RPL10A/uL1 in gene expression, we next performed ribosome profiling^29^ on wild-type and *Rpl10a* ^LOF/LOF^ embryos (*n* = 3 each). To identify direct targets of RPL10A/uL1-mediated regulation, we employed embryos at E8.5, an early stage in embryonic development when wild-type and *Rpl10a* ^LOF/LOF^ embryos are indistinguishable. In parallel, we also performed RNA-seq from these same embryos. The ribosome-protected footprint (RPF) ribosome profiling libraries had the expected enrichment for gene coding sequences (CDS) compared to the RNA-seq libraries (RNA), and the correlation between replicates was high (Supplementary Fig. 11). By comparing the RNA and RPF sequencing, we can identify genes that change at the level of transcript abundance, at the level of ribosome occupancy, or coordinately at both levels. We identified several hundred transcripts in each of these categories, including approximately 700 transcripts that change in their ribosome occupancy without changing significantly in mRNA abundance, suggesting that their translation may be regulated by RPL10A/uL1 (Fig. 6c and Supplementary Data 2). Genes with decreased translation in *Rpl10a* ^*LOF/LOF*^ embryos had significantly higher GC content in their 5’ untranslated regions (5’UTRs) (Supplementary Fig. 12). To determine whether RPL10A/uL1 is selectively regulating specific gene ontology groups, we calculated the translation efficiency for each gene and used these values for gene set enrichment analysis using CAMERA^30^. We found that transcripts with altered translation efficiency were significantly (false discovery rate (FDR) < 0.1) enriched for both the canonical and noncanonical Wnt signaling pathways (Fig. 6d and Supplementary Data 3). To further identify targets of RPL10A/uL1-mediated translational regulation, we examined these gene sets for transcripts that had significant (FDR < 0.1) changes in ribosome occupancy but not in total RNA abundance (Fig. 6e) and chose to further evaluate *Vangl2* and *Fzd3* (Supplementary Fig. 13). *Vangl2* is one of the core components of the noncanonical Wnt/planar cell polarity (PCP) pathway, whose asymmetric membrane localization is critical for establishing directionality across a tissue plane^31^. *Fzd3* is a receptor for Wnt ligands that, in addition to canonical Wnt signaling functions, also regulates the establishment of planar cell polarity^32,33^. These core developmental signaling pathways have well-established roles in regulating mesoderm differentiation and axis elongation, and notably, the *Rpl10a* ^LOF/LOF^ paraxial mesoderm defects are similar to the phenotypes caused by disruptions to noncanonical Wnt signaling^34,35^. To confirm that translation of *Vangl2* and *Fzd3* is decreased in *Rpl10a* ^LOF/LOF^ embryos, we performed sucrose gradient fractionation on wild-type and *Rpl10a* ^LOF/LOF^ E9.5 embryos and RT-qPCR to quantify the abundance of these mRNAs in each gradient fraction. We observed a significant shift towards the lighter, less translated fractions in *Rpl10a* ^LOF/LOF^ embryos for *Vangl2* (Fig. 6f) and *Fzd3* (Supplementary Fig. 14) but not for the control transcripts *Gapdh* and *Actb* (Supplementary Fig. 14), confirming that *Vangl2* and *Fzd3* are less translated in *Rpl10a* ^LOF/LOF^ embryos. These findings suggest that RPL10A/uL1-mediated translation may provide a novel regulatory layer to Wnt signaling networks to tune the production of paraxial mesoderm.

**Fig. 7:**
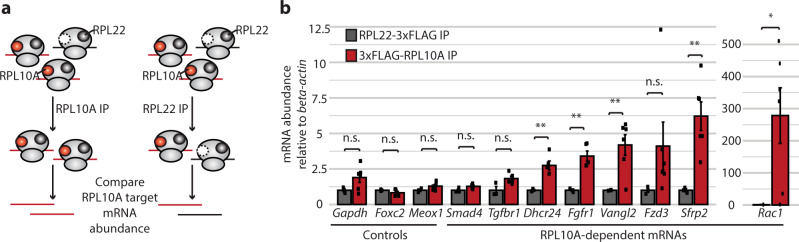
Wnt pathway mRNAs are enriched on RPL10A/uL1-containing ribosomes. **a** Schematic of 3xFLAG-RPL10A/uL1 and RPL22/eL22-3xFLAG ribosome immunoprecipitation from in vitro differentiated sclerotome and measurements of association with target mRNAs by RT-qPCR. **b** Genes identified as differentially translated in *Rpl10a* ^*LOF/LOF*^ embryos showed increased association with RPL10A-uL1-containing ribosomes compared to control transcripts that were unchanged in the ribosome profiling. Data shown are mean +/− SEM. Student’s *t* test *P* values: 0.05 (*Gapdh*), 0.11 (*Foxc2*), 0.07 (*Meox1*), 0.08 (*Smad4*), 0.06 (*Tgfbr1*), 0.005 (*Dhcr24*), 0.002 (*Fgfr1*), 0.007 (*Vangl2*), 0.12 (*Fzd3*), 0.003 (*Sfrp2*), 0.02 (*Rac1*). *P value < 0.05; **P value < 0.01. Source data are provided as a Source Data file.

To directly test whether there is an enrichment of Wnt pathway mRNAs on RPL10A/uL1-containing ribosomes, we used CRISPR/Cas9 to stably integrate a transgene expressing 3xFLAG-tagged RPL10A/uL1 at the *AAVS1* safe-harbor locus in hESCs. As a control, we also created a transgenic hESC line expressing RPL22/eL22-3xFLAG, which is not heterogeneous in its ribosomal incorporation during hESC differentiation and has been demonstrated to have no preferential translation of specific mRNA transcripts in mESCs^8^. This permitted us to pull down these two ribosomal populations—those containing RPL10A/uL1 or those containing RPL22/eL22 (which in turn would be a mixture of ribosomes containing and lacking RPL10A/uL1)—and determine the relative abundance of Wnt pathway mRNAs (Fig. 7a and Supplementary Fig. 15A). We performed this experiment in in vitro differentiated sclerotome, where RPL10A/uL1 has its lowest polysomal abundance, in order to maximize the contrast between 3xFLAG-RPL10A/uL1 and RPL22/eL22-3xFLAG ribosomal populations. The sclerotome was treated with the drug cycloheximide to freeze actively translating ribosomes onto their mRNAs, and a sucrose cushion was performed to isolate fully assembled ribosomal subunits for use as the input for immunoprecipitation (Supplementary Fig. 15A). Western blot analysis of the 3xFLAG-RPL10A/uL1 or RPL22/eL22-3xFLAG immunoprecipitations indicated successful isolation of ribosomes containing these tagged ribosomal proteins (Supplementary Fig. 15B). As cycloheximide treatment would stabilize all elongating ribosomes on the same mRNA molecule, regardless of whether or not they contain a tagged RP, a small number of untagged ribosomes were also present in the elution, but we confirmed this was due to tagged and untagged ribosomes forming a common polysome because RNase treatment to separate polysomes into individual monosomes resulted in the isolation of pure populations of 3xFLAG-RPL10A/uL1 or RPL22/eL22-3xFLAG containing ribosomes (Supplementary Fig. 15C).

Once 3xFLAG-RPL10A/uL1-containing or RPL22/eL22-3xFLAG-containing ribosomes were isolated, we examined the abundance of multiple mRNAs that had reduced translation in *Rpl10a* ^*LOF/LOF*^ embryos as well as control mRNAs that showed no change in translation in the ribosome profiling. While control mRNAs showed similar pull-down efficiencies between 3xFLAG-RPL10A/uL1 and RPL22/eL22-3xFLAG immunoprecipitations, mRNAs with reduced translation in *Rpl10a* ^*LOF/LOF*^ embryos, including Wnt pathway mRNAs, were enriched in 3xFLAG-RPL10A/uL1 immunoprecipitations compared to RPL22/eL22-3xFLAG (Fig. 7b). This indicates that Wnt pathway mRNAs are preferentially associated with RPL10A/uL1-containing ribosomes and suggests that RPL10A/uL1 may directly recruit target mRNAs to promote their translation.

### *Rpl10a*^LOF/LOF^ embryos have decreased canonical Wnt and PCP signaling

**Fig. 8:**
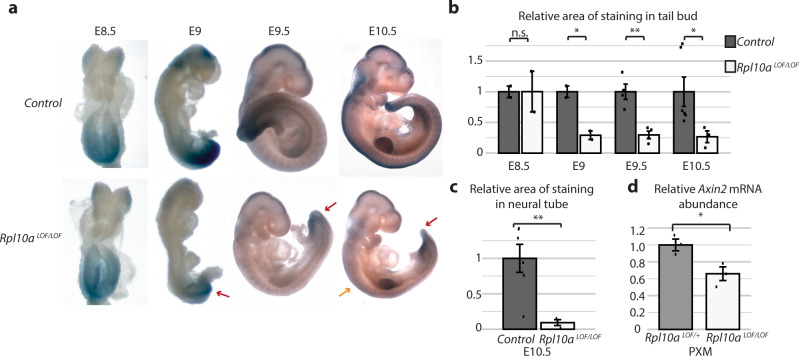
*Rpl10a*^*LOF/LOF*^ embryos have reduced canonical Wnt signaling. **a** Whole-embryo X-gal staining of *Axin2* ^LacZ/+^; *Rpl10a* ^+/+^ and *Axin2* ^LacZ/+^; *Rpl10a* ^LOF/LOF^ embryos. Decreased staining in *Axin2* ^LacZ/+^; *Rpl10a* ^LOF/LOF^ tail buds is indicated with red arrows; decreased staining in *Axin2* ^LacZ/+^; *Rpl10a* ^LOF/LOF^ neural tube is indicated with an orange arrow. **b** Quantification of the area of X-gal staining in tail bud relative to overall tail bud area. E8.5 and E9 *n* = 2 each, E9.5 *n* = 4, E10.5 *n* = 3 for *Rpl10a* ^*LOF/LOF*^ and *n* = 6 for control. Data are shown as mean +/− SEM. Student’s *t* test *P* values: 0.9 (E8.5), 0.03 (E9), 0.006 (E9.5), 0.03 (E10.5). **c** Quantification of the area of X-gal staining in the neural tube relative to overall neural tube area in E10.5 embryos (*n* = 3 for *Rpl10a* ^*LOF/LOF*^ and *n* = 6 for control). Data are shown as mean +/− SEM and significance was calculated using Student’s *t* tests (*P* = 0.005). **d** Abundance of *Axin2* mRNA relative to *Nupl1* control transcript as measured by RT-qPCR in in vitro differentiated paraxial mesoderm derived from heterozygous and homozygous *Rpl10a* ^*LOF/LOF*^ hESCs (*n* = 3 each). Data are shown as mean +/− SEM, and significance was calculated using Student’s *t* tests (*P* = 0.03). *P value < 0.05; **P value < 0.01. Source data are provided as a Source Data file.

To determine whether the decreased translation of Wnt pathway components results in decreased Wnt signaling in the embryo, we crossed the *Rpl10a* ^LOF^ mice with the *Axin2* β-galactosidase knockin reporter line (*Axin2* ^lacZ^), which allows tissues with active canonical Wnt signaling pathways to be visualized with X-gal staining^36^. Wnt signaling is indistinguishable in E8.5 wild-type and *Rpl10a* ^LOF/LOF^ embryos, but beginning at E9 and persisting through E10.5, the *Rpl10a* ^LOF/LOF^ embryos show reductions in X-gal staining particularly at the tail bud (Fig. 8a, red arrows, and Fig. 8b). In addition, at E10.5 we noted decreased X-gal staining in the neural tube of *Rpl10a* ^LOF/LOF^ embryos (Fig. 8a, orange arrow, and Fig. 8c), but staining in the limb buds did not differ across genotypes (Supplementary Fig. 16A). We similarly observed decreased *Axin2* mRNA expression in paraxial mesoderm derived from *Rpl10a* ^*LOF/LOF*^ hESCs, indicating that canonical Wnt signaling was also altered upon mutation of *Rpl10a* in our in vitro differentiation system (Fig. 8d). Notably, expression of the extended loss-of-function RPL10A/uL1 as a transgene from the *AAVS1* locus in hESCs, which results in increased expression of the extended RPL10A/uL1 compared to the endogenously edited *Rpl10a* ^*LOF/+*^ hESC cell line (Supplementary Fig. 16B), does not reduce *Axin2* expression upon paraxial mesoderm induction (Supplementary Fig. 16C), suggesting that the N-terminal extension of RPL10A/uL1 does not have a dominant negative effect upon canonical Wnt signaling.

**Fig. 9:**
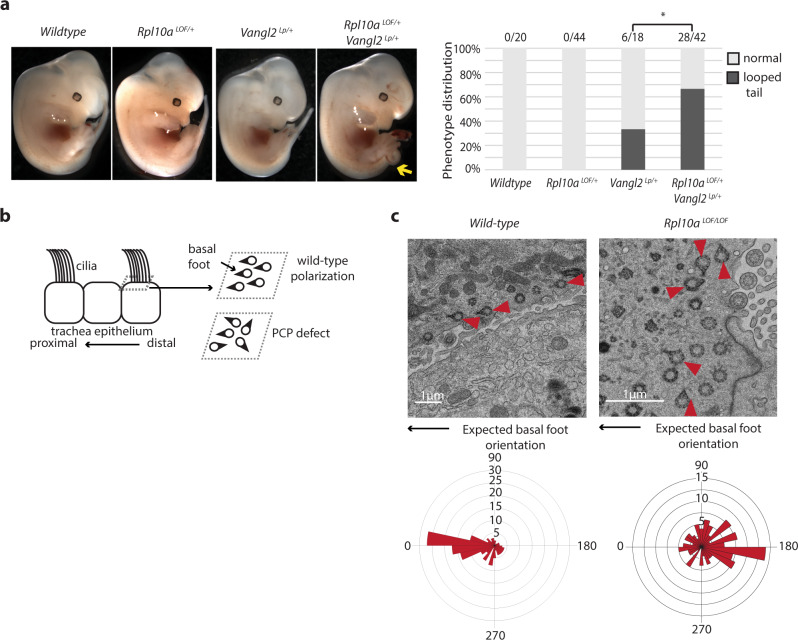
*Rpl10a*^*LOF/LOF*^ embryos have reduced noncanonical Wnt/planar cell polarity signaling. **a** Lateral views of E12.5 wild-type, *Rpl10a* ^*LOF*/+^, *Vangl2* ^*Lp*/+^, and *Rpl10a* ^*LOF*/+^; *Vangl2* ^*Lp*/+^ embryos and graph of looped tail phenotype frequencies for each genotype. The *Rpl10a* ^*LOF*/+^; *Vangl2* ^*Lp*/+^ embryo shown has the looped tail phenotype (arrow). The increased frequency in *Rpl10a* ^*LOF*/+^; *Vangl2* ^*Lp*/+^ embryos is statistically significant (*P* = 0.02, Fisher’s exact test). **b** Schematic of basal foot polarization in the multiciliated cells of the trachea epithelium. Basal feet point in the proximal direction (toward the larynx) in wild-type embryos and are less uniformly oriented when PCP signaling is perturbed. **c** Transmission electron microscopy of wild-type and *Rpl10a* ^*LOF*/LOF^ E18.5 trachea with basal bodies with visible basal feet demarcated with red arrowheads. Basal feet orientation relative to proximal–distal axis of the trachea, with the oral direction set to 0°, are shown in rose plots with wild-type *n* = 151 and *Rpl10a* ^*LOF*/LOF^ *n* = 127. The circular grid lines represent frequencies, and the difference in distribution between wild-type and *Rpl10a* ^*LOF*/LOF^ is statistically significant (Watson’s *U*^2^ test *P* value < 0.001). *P value < 0.05; **P value < 0.01. Source data are provided as a Source Data file.

As the PCP pathway also has known roles in embryonic axis elongation, we further sought to determine if *Rpl10a* controls PCP. We crossed the *Rpl10a* ^LOF^ mice with the *Vangl2* Loop-tail (Lp) mutant line, a well-established mouse line with defects in PCP signaling^37,38^, and collected embryos at mid-gestation. *Vangl2* ^Lp/+^ mice exhibit the eponymous looped tail phenotype at incomplete penetrance, but the frequency of the looped tail is doubled in *Vangl2* ^Lp/+^; *Rpl10a* ^LOF/+^ double heterozygotes (p = 0.02, Fisher’s exact test) (Fig. 9a). At low frequencies *Vangl2* ^Lp/+^ mice can also present with spina bifida, an incomplete closure of the neural tube. This phenotype was also present at double the frequency in *Vangl2* ^Lp/+^; *Rpl10a* ^LOF/+^ double heterozygotes compared to *Vangl2* ^Lp/+^ embryos, though it did not achieve statistical significance due to its overall rarity (Supplementary Fig. 16D). Notably, performing the same genetic interaction experiment with *Vangl2*^*Lp*^ and *T*^*Cre*^*; Rps6*^*lox*^ did not result in an increased incidence of the looped tail phenotype, indicating that *Vangl2* genetically interacts with *Rpl10a* but not *Rps6* (Supplementary Fig. 16E). We further assessed PCP pathway activity in E18.5 *Rpl10a* ^LOF/LOF^ embryos by examining the orientation of the basal feet on the basal bodies of cilia on the multiciliated cells (MCCs) of the trachea epithelium. The PCP signaling pathway ensures that cilia are aligned along the proximal–distal axis of the airway^39^ (Fig. 9b). Using transmission electron microscopy, we measured the angle of the basal foot appendage on each basal body relative to the axis of the trachea. While wild-type embryos showed the expected basal feet orientation in the direction of the larynx (set to 0°), *Rpl10a* ^LOF/LOF^ embryos were less polarized (Watson’s *U*^2^ test *P* < 0.001) (Fig. 9c). These findings reveal that RPL10A/uL1 modulates both the canonical and noncanonical arms of the Wnt signaling pathway.

## Discussion

Together, our studies identify a surprising change in ribosome composition that is observed during hESC differentiation. The considerable heterogeneity and rapid remodeling of ribosome composition during hESC differentiation suggest extensive, multilayered regulation of ribosome composition that is attuned to cell fate. Therefore, RPs may exert more specialized functions in control of mRNA translation as exemplified by RPL10A/uL1. Identifying these unique functions has historically been challenging. Our *Rpl10a* loss-of-function allele may serve as an important means to uncouple the diverse cellular roles of additional RPs: by engineering RP alleles that are incorporated into the ribosome, yet produce loss-of-function phenotypes, the housekeeping functions of RPs can be uncoupled from specialized roles in the cytoplasm.

The biochemical mechanism by which the RPL10A/uL1 N-terminal extension impedes RPL10A/uL1 function has yet to be elucidated. RPL10A/uL1 is found at the base of the L1 stalk, a highly flexible region of ribosomal RNA that regulates tRNA exit during translation elongation as well as serving as a binding platform for ribosome biogenesis factors and mRNA surveillance pathway components^40,41^. While potentially contributing to L1 stalk function, RPL10A/uL1 has also been shown to directly contact viral internal ribosome entry site (IRES) elements^42^ and promote their translation^8^, indicating that RPL10A/uL1 may be a multifaceted protein with both general and specialized translation activities. Indeed, our findings that Wnt pathway mRNAs are enriched on RPL10A/uL1-containing ribosomes suggest that RPL10A/uL1 may be capable of direct recruitment of target mRNAs. Our observation that genes with decreased translation in *Rpl10a* ^LOF/LOF^ embryos tend to have greater GC content in their 5’UTRs suggests that they may have stable secondary structures within their 5’UTRs that could serve as points of regulation. This is in keeping with a previous report of preferential translation of specific mRNAs, including several with known IRES-like elements, by RPL10A/uL1-containing ribosomes in mESCs^8^. Although Wnt signaling was not an enriched gene ontology term in that study, this is likely due to the differences in gene expression programs between mESCs and E8.5 mouse embryos. As the loss-of-function extension is at the solvent-exposed N-terminus, it may inhibit translation of Wnt pathway components and other RPL10A/uL1-dependent mRNAs by preventing RPL10A/uL1 from binding to these transcripts directly or from associating with additional *trans*-acting factors.

The specific triggers and the molecular mechanisms underlying changes in ribosome composition reflect a new frontier of study. While our roadmap of ribosome composition changes has revealed the magnitude and rapidity of ribosome remodeling, it is not yet possible to determine the precise number of possible ribosome compositions without first identifying whether the presence or absence of these heterogeneous ribosomal proteins are independent events or coordinately regulated. These changes to ribosome composition may occur within the framework of the canonical process of ribosome biogenesis, but our careful quantification of RP composition dynamics in the nucleus, cytoplasm, and across ribosome subunits also suggests the intriguing possibility for exchange of RPs on and off of assembled cytoplasmic ribosomal subunits. RP exchange has been shown to occur on ribosomes within neuronal processes^43,44^, but it remains to be determined whether this is also a mechanism of remodeling the ribosome in response to differentiation cues.

It is striking that a single, ancient RP such as RPL10A/uL1 simultaneously regulates the translation of multiple components of the Wnt signaling pathway. This suggests a longstanding partnership between the ribosome and core signaling networks in control of cell fate and embryonic development. Given the extensive and long-studied transcriptional and post-translational regulation of Wnt signaling, the developmental importance of this new layer of translational control, as revealed by the prominent phenotypes of the *Rpl10a* ^*LOF/LOF*^ mouse model, may represent a useful mechanism for tissue-specific fine-tuning of Wnt signaling. Indeed, large portions of the mouse embryonic transcriptome, including such core developmental networks as the Wnt and Shh pathways, are translated at different rates across tissues^45^, though whether these patterns are directly mediated by changes in ribosome composition remain to be determined. Additionally, the centrality of Wnt signaling to not only embryogenesis but also adult tissue maintenance and cancer raises the question of whether RPL10A/uL1-mediated translational control of Wnt signaling components may also play important roles in these processes. Taken together, our findings highlight the modularity of ribosome composition and the critical, previously underappreciated functions for RPs in control of cell signaling and embryonic development.

## Methods

### Research ethics statement

All human embryonic stem cell work was conducted with the approval of the Stanford University Stem Cell Research Oversight (SCRO) committee. All animal husbandry and experiments were performed with the approval of the Stanford University Administrative Panel on Laboratory Animal Care (APLAC).

### hESC cell culture and in vitro differentiation

H7 hESCs were cultured in mTeSR1 media (StemCell Technologies) on plastic dishes coated with Geltrex (Gibco). hESCs were passaged using Accutase (Gibco) and cultured overnight in mTeSR1 supplemented with 1 µM thiazovivin (Tocris) to promote cell survival. For each cell differentiation experiment, hESCs were grown in parallel to serve as control samples. hESCs were differentiated as previously described^10,11^: in brief, cells were washed in DMEM/F12 before addition of the CDM2 basal differentiation media (50% IMDM (+GlutaMAX, +HEPES, + sodium bicarbonate; Gibco, 31980-097) + 50% F12 (+GlutaMAX; Gibco, 31765-092) + 1 mg/mL polyvinyl alcohol (Sigma, P8136-250G) + 1% v/v concentrated lipids (Gibco, 11905-031) + 450 μM monothioglycerol (Sigma, M6145) + 0.7 μg/mL insulin (Roche, 1376497) + 15 μg/mL transferrin (Roche, 652202) + 1% v/v penicillin/streptomycin (Gibco)) containing the appropriate growth factors. For hESC induction into APS: 30 ng/mL Activin A (R&D 338-AC-050) + 4 μM CHIR99021 (Tocris 4423) + 20 ng/mL FGF2 (R&D 233-FB-01M) + 100 nM PIK90 (EMD Millipore 528117-5MG). For APS induction into PXM: 1 μM A-83-01 (Tocris 2939) + 3 μM CHIR99021 + 250 nM LDN-193189 (Stemgent 04-0074) + 20 ng/mL FGF2. For PXM induction in ESom: 1 μM A-83-01 + 250 nM LDN-193189 + 1 μM XAV939 (Tocris 3748) + 100 nM AZD4547 (Cellagen C2454-5S). For ESom induction into sclerotome: 5 nM 21 K + 1 μM C59 (Cellagen C7641-2S). For hESC induction into AmPS: 100 ng/mL Activin A + 3 μM CHIR99021 + 20 ng/mL FGF2 + 50 nM PI-103 (Tocris 2930). For AmPS induction into DE: 100 ng/mL Activin A + 250 nM LDN-193189 + 20 ng/mL FGF2. For DE induction into MHG: 10 ng/mL BMP4 (R&D 314-BP-050) + 3 μM CHIR99021 + 100 ng/mL FGF2.

To confirm proper hESC differentiation into the target cell types, RNA was extracted from each cell type using TRIzol (Thermo Fisher) and the Direct-zol Micro Kit (Zymo) followed by DNase digest with TURBO DNase at 37 °C for 30 min. DNase was removed using the RNA Clean and Concentrator-5 kit (Zymo). In total, 200 ng RNA was used for reverse transcription using the iScript Supermix (Bio-rad). The cDNA was diluted 20-fold, and 4 µl used as the template for qPCR with SsoAdvanced SYBR Green supermix (Bio-Rad) on a CFX384 machine (Bio-rad). Undetermined Ct values were assigned a value of 40 as a conservative overestimate of gene expression. The expression of each gene was normalized to the expression of a housekeeping gene (*Nupl1)*. Primer sequences are provided in Supplementary Table 1.

### Cell lysis and fractionation

For cytoplasmic extraction, hESCs and differentiated cells were harvested with Accutase, and the cell pellets were lysed in lysis buffer (20 mM Tris pH 7.5, 150 mM NaCl, 15 mM MgCl_2_, 100 µg/ml cycloheximide, 1 mM DTT, 0.5% Triton X-100, 0.1 mg/ml heparin, 8% glycerol, 20 U/ml TURBO DNase (Ambion, AM2238), 200 U/mL SUPERase In RNase Inhibitor (Ambion, AM2696), 1× Combined Protease and Phosphatase Inhibitor (Thermo Scientific, 78443)) at 4 °C for 30 min with occasional vortexing. Lysates were sequentially centrifuged at 1800×*g* for 5 min at 4 °C and then at 10,000×*g* for 5 min at 4 °C, retaining the final supernatant as the cytoplasmic extract. To collect polysomes, cytoplasmic extract was loaded onto a 10–45% sucrose gradient (20 mM Tris pH 7.5, 100 mM NaCl, 15 mM MgCl_2_, 100 μg/ml cycloheximide, made on a Biocomp Model 108 Gradient Master) and centrifuged in a Beckman SW41 rotor at 40,000 rpm for 2.5 h at 4 °C in a Beckman L8-80M ultracentrifuge. Gradients were then fractionated on a Density Gradient Fraction System (Brandel, BR-188) using PeakChart software (v1.02) with continuous A_260_ measurements. Fractions corresponding to three or more ribosomes per transcript were considered polysomal, and protein was extracted using the Proteoextract Protein Precipitation Kit (Calbiochem) following kit instructions. Precipitated protein was resuspended in 20 mM Tris pH 7.5, 50 mM KOAc, 10% glycerol, 1 mM DTT. Cytoplasmic extract and purified polysomal protein concentrations were measured by Bradford assay, and equal quantities (at least 10 µg each) of hESC and differentiated cell material were used for all downstream mass spectrometry sample preparation.

For whole-cell lysate preparation, cells were lysed using the Pierce Mass Spec Sample Kit for Cultured Cells, following the manufacturer’s protocol. Lysate concentrations were calculated using the BCA protein assay kit (Thermo Scientific), and equal amounts of protein from hESC and differentiated cells were used for all downstream sample preparation steps.

### TMT sample preparation and mass spectrometry

For cytoplasmic extract and polysome samples, protein was denatured in 50 mM ammonium bicarbonate, 2 M urea, 5 mM DTT at 65 °C for 1 h, followed by alkylation with 15 mM iodoacetamide at room temperature protected from light. Samples were digested with a 50:1 protein to trypsin (Pierce) mass ratio at 37 °C overnight and desalted using OMIX C18 pipet tip columns (Agilent A57003100) according to the manufacturer’s instructions and dried with a Speed Vac. For whole-cell lysates, reduction, alkylation, and digestion were performed using the Pierce Mass Spec Sample Kit for Cultured Cells, following the manufacturer’s protocol.

Dried peptides were resuspended in 20 mM HEPES pH 8.0 (AppliChem) and labeled with unique TMT labels from the TMT sixplex isobaric label reagent set (Thermo Scientific) following the manufacturer’s instructions. Reactions were quenched and equal masses of each labeled sample were combined and acidified with heptafluorobutyric acid (HFBA) to a final concentration of 0.5% volume/volume and formic acid to a final concentration of 5% volume/volume. Samples were desalted with OMIX C18 pipet tip columns, dried on a Speed Vac, and resuspended in 15 µl 5% acetonitrile + 0.1% volume/volume trifluoroacetic acid. In all, 3 µl of each sample was loaded onto the HPLC (Waters) using a 20-25 cm HPLC column packed with 2.7 µm C18 resin (Halo), a flow rate of 0.3 µl/min, and a 180 min gradient from 95% A (0.1% formic acid in HPLC-grade water) 5% B (0.1% formic acid in acetonitrile) to 60% A 40% B. Acquisition was performed on an Orbitrap Elite mass spectrometer (Thermo Scientific) using Xcalibur (Thermo Scientific, v4.1). Analysis was performed using Proteome Discoverer 1.4 (Thermo Scientific) using the Mascot search engine, using only unique peptides for protein quantification and a high confidence filter of peptide FDR < 0.01. The number of peptides used to calculate the abundance of each RP is listed in Supplementary Data 1: on average, eight peptides were used to identify each ribosomal protein, and the quantification between peptides for each RP was typically consistent, with a median standard deviation between peptides of 0.15. On the rare occasion that an RP was not successfully identified in a mass spectrometry sample, no imputation was used to replace the missing value. The ratio of the abundance of each RP in the hESCs relative to the differentiated cell sample was normalized by the median relative abundance of all RPs within the respective subunit (40 S or 60 S) to account for any differences in TMT labeling efficiencies or differences in the total amount of ribosomal subunits in each cell type. At least five whole-cell lysate and at least six cytoplasmic extract and polysome replicates were performed for each differentiated cell type, and the values given in the manuscript are the medians of these replicates. The protein-level quantification values were used for statistical analysis with ANOVA to assess the significance of the relative abundance changes of each RP over the course of cell differentiation. All data used for ANOVA analysis, and the resulting *P* values, are listed in Supplementary Data 1.

### Structural analysis

Models of the human ribosome (PDB: 4v6x) were downloaded from the Protein Data Bank (PDB) website and edited using PyMOL (v2.3.3). The fraction of solvent-accessible surface area for each RP was calculated by determining the total surface area and subtracting all interface areas (from interactions with other RPs and rRNA), which were calculated using PDBePISA (v1.48).

### RNA-seq analysis of RP expression

Default parameters were used for computational tools except where noted otherwise. Reference genome GRCh38.p13 (primary assembly) was used with GENCODE version 36 annotations. Yale-UCSC two-way consensus annotations for pseudogenes (gencode.v36.2wayconspseudos.gtf.gz) were filtered for entries in which RP genes were listed as the parent gene, yielding 2024 pseudogenes. Reference sequences for ERCC92 standards (Thermo Fisher) were concatenated to the reference genome. RP pseudogene sequences were hard-masked using the bedtools maskfasta (version 2.29.2) command^46^. The reference genome for STAR (version 2.7.6a) was generated with sjdbOverhang=149. The reference genome for RSEM (version 1.3.3) was prepared according to default parameters.

Raw reads (.fastq.gz) were downloaded from the NIH Gene Expression Omnibus according to the run IDs listed below. Adapters were trimmed using Skewer (version 0.1.127) with the following parameters: for mesoderm, “-x AGATCGGAAGAGCACACGTCTGAACTCCAGTCACNNNNNNATCTCGTATGCCGTCTTCTGCTTG -y AGATCGGAAGAGCGTCGTGTAGGGAAAGAGTGTAGATCTCGGTGGTCGCCGTATCATT -t 1 -q 21 -l 21 -n -u -f sanger -z”; for endoderm, “-t 1 -m any -q 21 -l 21 -n -u -z”.

Alignment was performed using STAR (version 2.7.6a) with the following parameters: “–outSAMunmapped Within–outFilterType BySJout–outSAMattributes NH HI AS NM MD–outFilterMultimapNmax 20–outFilterMismatchNmax 999–outFilterMismatchNoverReadLmax 0.01–alignIntronMin 20–alignIntronMax 1000000–alignMatesGapMax 1000000–alignSJoverhangMin 8–alignSJDBoverhangMin 1–sjdbScore 1–readFilesCommand zcat–twopassMode Basic–twopass1readsN −1–limitBAMsortRAM 60000000000–outSAMtype BAM SortedByCoordinate–quantMode TranscriptomeSAM”. Quantification was performed using rsem-calculate-expression (version 1.3.3) with the following parameters: “–bam–estimate-rspd–no-bam-output–seed 12345”. For the mesoderm samples, the additional parameters “–paired-end–forward-prob 0” were included.

For mesoderm samples, transcripts per million (TPM) values were normalized by the mean TPM of each gene among the hESC replicates. In endoderm samples, TPM values were normalized by the TPM of each gene in the hESC sample. Within each sample, each RP gene’s expression was normalized by the median expression value among RPs in the respective subunit (i.e., RPS3 was normalized by the median expression of 40 S RPs).

### Creation of *Rpl10a* mouse models

The conditional *Rpl10a* deletion allele was created using a targeting vector purchased from EUCOMM (MGI allele Rpl10a^tm1a(EUCOMM)Hmgu^). Removal of the selection cassettes was performed by crossing with the ROSA26::FLPe knock in line (JAX #009086), and the deletion was accomplished by crossing with CMV-Cre line (JAX #006054). Cre recombination was confirmed by genomic DNA qPCR using SsoAdvanced SYBR Green supermix (Bio-Rad, catalog no. 1725270) on a CFX384 machine (Bio-rad). Mice were maintained on a C57BL/6 background. Genotyping of the conditional allele was performed using standard PCR protocols with the following primers: OS127 (caagaaacagctacagtggctt) and OS349 (cataacgataccacgatatcaaca), expected band size 1.5 kb for conditional allele and 500 bp for deletion allele.

The null and extended Rpl10a alleles were generated by injection of a gRNA (target sequence AACAACTTACCTCATGGCTG) targeting the Rpl10a locus into C57BL/6 mouse embryos, which was performed by the UCSF Gladstone Transgenic Core Facility. Mice were maintained on a C57BL/6 background and genotyped using the following primers: OS57 (AAACCGCTCACTTGCGGCGCTTGC) and OS60 (AGCAGGGAGAAATCCAATCC), expected band size 534 bp for wild-type allele, 456 bp for extended allele, and 441 bp for null allele.

Quantification of posterior trunk length was performed on lateral embryo images using ImageJ. For E8.5 embryos, this was measured as the distance from the posterior tip of the embryo to the inflection point normalized to the distance from the inflection point to the anterior tip of the embryo. For E9.5 embryos, this was measured as the distance from the forelimb bud to the posterior tip of the tail bud normalized to the distance from the forelimb bud to the otic placode. For E10.5-E12.5 embryo, this was measured as the distance from the hindlimb bud to the posterior tip of the tail bud normalized to the distance between the fore- and hindlimb buds.

### Mouse husbandry

All mouse work was reviewed and approved by the Stanford Administrative Panel on Laboratory Animal Care (APLAC). Animals were housed in standard conditions with 12-h light/dark cycles, ambient temperatures between 68 and 79 °F, and humidity between 30 and 70%. The following previously published mouse lines were used: ROSA26::FLPe (JAX #009086), CMV-Cre (JAX #006054), Meox1 Cre^28^, Ai9 (JAX #007909), T Cre^27^, Rps6^lox^^22^, Axin2^lacZ^ (JAX #009120), and Vangl2^Lp^ (JAX # 000220). Genotyping was performed using standard DNA isolation and PCR protocols using the primers described in the cited publications or on the Jackson Laboratory website. Primer sequences are available in Supplementary Table 1. To generate embryos, a male was housed with one or two females and the females monitored for vaginal plug formation. On the day a plug was observed, the female was considered to be pregnant at E0.5. Embryos were harvested from the pregnant female at E8.5, E9.5, E10.5, E12.5, E14.5, E17.5, or E18.5, depending on the experiment. The biological sex of the embryos was not determined.

### Embryo RNA isolation and RT-qPCR

RNA was extracted from embryos in TRIzol (Thermo Fisher) following the manufacturer’s instructions. cDNA was made using 200 ng RNA iScript Supermix (Bio-rad) and qPCR performed with SsoAdvanced SYBR Green supermix (Bio-Rad, catalog no. 1725270) on a CFX384 machine (Bio-rad). All primers are listed in Supplementary Table 1.

### In situ hybridization

Digoxigenin (DIG)-labeled probes were synthesized, and E9.5 and E10.5 embryos used for whole-mount in situ hybridization following standard methods^47,48^. Embryos were fixed in 4% paraformaldehyde, washed twice with PBST, and dehydrated through a methanol series. Embryos were rehydrated through 75%, 50%, 25% methanol in PBST and then washed twice in PBST before treating with 5 µg/mL proteinase K in PBST at room temperature for 15 min for E9.5 stage embryos or 20 min for E10.5 stage embryos. Embryos were then washed with PBST, post-fixed in 4% paraformaldehyde + 0.1% glutaraldehyde in PBST at room temperature for 20 min, washed with PBST, and equilibrated to hybridization mix (50% formamide, 1.3× SSC, 5 mM EDTA, 50 µg/mL yeast RNA (Sigma R-6625), 0.2% Tween-20, 0.5% CHAPS, 100 µg/mL heparin). Embryos in the hybridization mix were incubated at 65°C in a hybridization oven with rocking for at least 1 h before the addition of 1 µg/mL DIG-labeled probes and incubated with rocking at 65 °C overnight. The following day the embryos were washed at 65 °C with hybridization mix, then a 1:1 mixture of hybridization and MABT (100 mM maleic acid, 150 mM NaCl, 0.1% Tween-20, pH 7.5), and then washed with MABT at room temperature. Blocking was performed at room temperature with 2% Boehringer Blocking Reagent in MABT for 1 h followed by 2% Boehringer Blocking Reagent + 20% heat-inactivated sheep serum in MABT for 1 h. AP-anti-DIG antibody (Roche 11093274910) at a 1:2000 dilution in 2% Boehringer Blocking Reagent + 20% heat-inactivated sheep serum in MABT was added to the embryos and incubated at 4 °C overnight. Embryos were washed at room temperature in MABT for an entire day and overnight, followed by two 20-min washes at room temperature in NTMT (100 mM NaCl, 100 mM Tris-HCl pH 9.5, 50 mM MgCl_2_, 0.1% Tween-20). Embryos were developed with BM Purple (Roche) at room temperature until adequate signal was achieved and then washed with PBST, re-fixed in 4% paraformaldehyde + 0.1% glutaraldehyde, and stored in PBST + 0.1% azide. Quantification was performed on lateral embryo images using ImageJ. For quantification of signal at the tail bud, a common signal intensity threshold across all genotypes was set, and a region of interest (ROI) set around the tail bud and paraxial mesoderm up to the location of the most posterior somite. The area of signal above the threshold within the ROI was calculated relative to the total area of the ROI. The distance between the posterior end of the notochord, as demarcated by *Shh* in situ signal, and the distal tip of the tail bud was measured in ImageJ and normalized by the distance between the forelimb bud and the otic placode on the same image.

### Skeletal and cartilage staining

Cartilage staining of E14.5 embryos was performed by fixing embryos in Bouin’s solution for 2 h at room temperature followed by 24 h of washes in 70% ethanol + 0.1% ammonium hydroxide (NH_4_OH). Embryos were then equilibrated by two 1-h incubations in 5% acetic acid. Embryos were stained with Alcian Blue (0.05% in 5% acetic acid) at room temperature for 2 h and washed twice with 5% acetic acid for 1 h each. Embryos were dehydrated by washing twice with methanol (1 h each) and then cleared in 1:2 benzyl alcohol/benzylbenzoate (BABB).

Cartilage and bone staining were performed on E17.5 embryos fixed in ethanol after evisceration and removal of the skin. Embryos were then incubated in acetone overnight, followed by staining with Alcian Blue (0.03% in 64% ethanol + 20% glacial acetic acid). Embryos were washed repeatedly with 70% ethanol, cleared with 1% potassium hydroxide (KOH), and counterstained with Alizarin Red (0.05% in 1% KOH). Embryos were cleared again with 1% KOH and taken through a glycerol series from 20% glycerol, 80% 1% KOH to 80% glycerol, 20% 1% KOH.

### hESC CRISPR

To introduce the loss-of-function insertion at the endogenous *Rpl10a* locus in H7 hESCs, a guide RNA (GCGCGGCGTGAGAAGCCATG) targeting the *Rpl10a* start codon was designed using Benchling, and the sgRNA synthesized using the Gene Art Precision gRNA Synthesis Kit (Thermo Fisher) according to kit instructions. In all, 10 µg of sgRNA was complexed with 16 µg of purified Cas9-NLS protein (UC Berkeley MacroLab) at room temperature for 5 min. The Cas9-sgRNA RNP was then nucleofected into 1 million hESCs along with 1 µL of a 100 µM ssODN containing the loss-of-function insertion and homology to the *Rpl10a* locus and 1.5 µg of pCE-mp53DD (Addgene #41856, to promote cell survival) using the Human Stem Cell Nucleofector Kit 2 (Lonza) following manufacturer’s instructions. Cells were plated in 1 well of a six-well plate post-nucleofection and cultured until confluency as sufficient to split. The clonal selection was then performed by sparse seeding of a single cell suspension in a 10-cm dish followed by manual picking of colonies into individual wells of a 96-well plate.

To introduce Flag-tagged RP transgenes into the *AAVS1* locus, 3xFLAG-RPL10A/uL1 or RPL22/eL22-3xFLAG coding sequences were cloned into an *AAVS1*-targeting vector (Addgene #107580, replacing the mTagRFP coding sequence) containing a CAGGS promoter and puromycin resistance cassette. *AAVS1*-targeting sgRNA was synthesized by using the Gene Art Precision gRNA Synthesis Kit (Thermo Fisher) according to kit instructions and complexed with purified Cas9-NLS protein as above. Two million H7 hESCs were nucleofected with the sgRNA-Cas9 RNP, 2.33 µg of the *AAVS1*-targeting vector, and 1.5 µg of pCE-mp53DD (Addgene #41856, to promote cell survival) using the Human Stem Cell Nucleofector Kit 2 (Lonza) following the manufacturer’s instructions and plated in one well of a six-well plate. Forty-eight hours post-nucleofection, the cells were treated with mTeSR1 + CloneR (StemCell Technologies) + 0.25 µg/mL puromycin for 48 h. Cells were then grown to confluency and clonally selected by sparse seeding as above.

### Cell viability assay

hESCs were passaged as described above, and cells were counted to ensure even plating across genotypes. Cells were plated in Geltrex-coated black-sided clear-bottomed 96-well tissue culture dishes, excluding the wells on the edge of the dish. Cell viability was measured using the CellTiter-Glo Luminescent Cell Viability Assay (Promega) following kit instructions. For viability measurements during in vitro differentiation, equal numbers of cells of each genotype were plated in each well, and half underwent differentiation while half were maintained as undifferentiated hESCs. The luminescence of the differentiated cells was then normalized to the luminescence of the undifferentiated hESCs for each genotype. Significance was measured using Student’s *t* tests.

### Embryo sucrose-gradient fractionation

Embryos were dissociated with 1% trypsin in Hanks’ Balanced Salt Solution (HBSS, Thermo Fisher, 14025-076) at 37 °C and the trypsin was neutralized with filming media (10% fetal bovine serum (FBS) in DMEM/F12 without phenol red). Cells were washed with PBS containing 100 µg/mL cycloheximide and lysed in lysis buffer (20 mM Tris pH 7.5, 150 mM NaCl, 15 mM MgCl_2_, 100 µg/ml cycloheximide, 1 mM DTT, 1% Triton X-100, 0.1 mg/ml heparin, 8% glycerol, 20 U/ml TURBO DNase (Ambion, AM2238), 200 U/mL SUPERase In RNase Inhibitor (Ambion, AM2696), 1× combined protease and phosphatase inhibitor (Thermo, 78443)) at 4 °C for 30 min with occasional vortexing, followed by sequential centrifugation at 1800×*g* for 5 min at 4 °C and then 10,000 ×*g* for 5 min at 4 °C. The clarified cytoplasmic extract was loaded onto 10–45% sucrose gradients (20 mM Tris pH 7.5, 100 mM NaCl, 15 mM MgCl_2_, 100 μg/ml cycloheximide, made on a Biocomp Model 108 Gradient Master) and centrifuged in a Beckman SW60 rotor at 35,000 rpm for 2.5 h at 4 °C in a Beckman L8-80M ultracentrifuge. Gradients were then fractionated on a Density Gradient Fraction System (Brandel, BR-188) with continuous A_260_ measurements using PeakChart software (v1.02). Protein from each individual fraction was extracted using the Proteoextract Protein Precipitation Kit (Calbiochem) following kit instructions and resuspended in 2× Laemmli buffer for western blot analysis.

### Western blot

Protein samples were run on 4–20% Tris-Glycine SDS gradient gels and transferred to PVDF membranes using the semi-dry Trans-Blot Turbo system (Bio-Rad). In all, 5% weight/volume milk in PBST was used for blocking and all antibody dilutions. Primary antibodies were incubated on the blots overnight at 4 °C, while horseradish peroxidase-coupled secondary antibodies were on blots for 1 h at room temperature. Clarity Western ECL Substrate (Bio-Rad, 1705060) was used for development, and blots were imaged on a ChemiDoc MP (Bio-Rad). Primary antibodies used: anti-RPL10A (Abcam ab174318, 1:1000 dilution), anti-RPL10A (Santa Cruz Biotechnology sc-100827, 1:1000 dilution), anti-RPL11 (Abcam ab79352, 1:1000 dilution), anti-RPL34 (Abcam ab129394, 1:500 dilution), anti-RPL23A (Bethyl A303-932A-M, 1:2000 dilution), anti-RPL22 (ProteinTech 250021AP), anti-RPS25 (Sigma HPA031801, 1:250 dilution), anti-RPS5 (Abcam ab58345, 1:1000 dilution), anti-GAPDH (Invitrogen AM4300, 1:2000 dilution), anti-β-actin (Cell Signaling 3700 S, 1:2000 dilution). Secondary antibodies used: donkey anti-mouse (GE Healthcare NA931-1ML, 1:5000 dilution) or donkey anti-rabbit (GE Healthcare NA934-1ML, 1:5000 dilution).

### O-propargyl-puromycin incorporation

To measure protein synthesis in mouse embryos, E9.5 embryos were dissociated with 1% trypsin in HBSS at 37 °C for 20 min. Embryos were washed once with filming media (10% FBS in DMEM/F12 without phenol red), and then the tissue was dissociated by repeated pipetting in filming media. A negative control embryo was treated with filming media + 100 µg/mL cycloheximide at 37 °C for 5 min prior to treatment with filming media + 100 µg/mL cycloheximide + 20 µM O-propargyl-puromycin (OPP); a second negative control sample was given filming media + DMSO; all other embryos were treated with filming media + 20 µM OPP for 30 min at 37 °C. Cells were washed with cold Dulbecco’s PBS (DPBS) and resuspended in Zombie Violet Live-Dead Stain (1:500 in DPBS; BioLegend, 423113). Cells were incubated at room temperature protected from light for 15 min, washed with cell staining buffer (0.1% NaN_3_, 2% FBS in HBSS), and fixed with 1% paraformaldehyde in DPBS on ice for 15 min. After fixation, the cells were washed with Perm buffer (0.1% Saponin, 0.1% NaN_3_, 3% FBS in PBS) and left in fresh Perm buffer at 4 °C overnight. The next day, cells were washed twice with 2% FBS in HBSS and then labeled with an Alexa Fluor 488 Picolyl Azide dye from the Click-iT Alexa Fluor Picolyl Azide Toolkit (Thermo Scientific, prepared following manufacturer’s instructions) for 30 min at room temperature in the dark. Labeled cells were washed and resuspended in cell staining buffer before being run on a Quanteon flow cytometer (Agilent) by the Stanford Shared FACS Facility and analyzed using the software package FlowJo.

To measure protein synthesis in hESCs, cells were washed with DMEM/F12 + glutamax (Gibco) and then given mTeSR1 containing 20 µM O-propargyl-puromycin (OPP) for 30 min at 37 °C. A negative control well was treated with mTeSR1 + 100 µg/mL cycloheximide at 37 °C for 5 min prior to treatment with mTeSR1 + 100 µg/mL cycloheximide + 20 µM OPP; a second negative control sample was given mTeSR1 + DMSO. Cells were then washed and harvested using Accutase (Gibco). The cell pellets were then washed with cold Dulbecco’s PBS (DPBS), stained with Zombie Violet, fixed, permeabilized, labeled with Alexa Fluor Picolyl Azide dye, and run on a flow cytometer as described above for embryos.

### Ribosome profiling

Whole E8.5 embryos were dissected in cold HBSS with 100 µg/mL cycloheximide, and each embryo was lysed for 30 min at 4 °C in 215 µL lysis buffer (20 mM Tris pH 7.5, 150 mM NaCl, 15 mM MgCl_2_, 1 mM DTT, 8% glycerol, 1% Triton X-100, 100 μg/ml cycloheximide, 20 U/ml Turbo DNAse (Thermo Scientific, AM2238), and Complete Protease Inhibitor EDTA-free (Sigma-Aldrich, 11836170001)). Lysates were sequentially centrifuged at 1300 ×*g* for 5 min at 4 °C and then 10,000×*g* for 10 min at 4 °C. In total, 70 µL of the clarified lysate was taken as the RNA input sample for RNA-seq and mixed with 55 µL water and 375 TRIzol LS (Thermo Scientific) and stored at −80 °C for subsequent RNA extraction. The remaining lysate was treated with 0.5 μg RNase A (Thermo Scientific, AM2271) and 300 U RNase T1 (Thermo Scientific, EN0541) for 30 min at room temperature with gentle rocking. These RNases were chosen to prevent degradation of the ribosome in the small amount of material present in a single E8.5 embryo. RNase activity was stopped by the addition of 100 U SUPERase RNase Inhibitor (Thermo Scientific, AM2694). Ribosomes were enriched by sucrose cushion (1 M sucrose in 20 mM Tris pH 7.5, 150 mM NaCl, 15 mM MgCl_2_, 1 mM DTT, 1× cOmplete Mini Protease Inhibitor Cocktail (Roche), 100 μg/ml cycloheximide, 20 U/mL SUPERase RNase Inhibitor), and centrifuging in a TLA 120.2 rotor (Beckman) at 70,000 rpm for 4 h at 4 °C in a Beckman TL-100 ultracentrifuge. The ribosome pellet containing the ribosome-protected Ribo-Seq library was resuspended in 500 μL TRIzol. Total RNA and ribosome footprints were extracted using the Direct-zol Micro Kit (Zymo) followed by DNase digest with TURBO DNase at 37 °C for 30 min. DNase was removed using the RNA Clean and Concentrator-5 kit (Zymo). The total RNA and ribosome-protected footprint samples were depleted of rRNA using Ribo-Zero Gold from the TruSeq Stranded Total RNA Library Prep Gold (Illumina 20020598) following the manufacturer’s instructions. The samples were then purified with the RNA Clean & Concentrator-5 kit (Zymo) with the modifications that 2 volumes of RNA Binding Buffer and 4.5 volumes of ethanol were added to ribosome footprinting samples to purify small RNAs and 1 volume of RNA Binding Buffer and 1 volume of ethanol was added to total RNA samples to isolate RNA > 200 nt. Total RNA samples were then diluted to 100 µL with 5 mM Tris-HCl pH 7.5, mixed with 100 µL of 2x alkaline fragmentation buffer (100 mM Na_2_CO_3_ pH 9.2, 2 mM EDTA), and incubated at 95°C for 20 min to partially fragment the RNA. The reaction was halted with 440 μL STOP Buffer (70 μL 3 M sodium acetate (NaOAc) pH 5.5, 2 μL Glycoblue, and 370 μL nuclease-free water) and precipitated with isopropanol overnight at −80 °C. Fragmented total RNA and ribosome-protected footprints were size-selected by running on a 15% tris-borate-EDTA (TBE)-urea polyacrylamide gel with 20/100 ladder (IDT 51-05-15-02) to identify the 30-70 nucleotide region (for total RNA) or the oNTI199 and oNTI265 oligos to demarcate the 28-34 nucleotide region (for ribosome-protected fragments). Gel slices were crushed and incubated at room temperature overnight in 400 μL RNA extraction buffer (300 mM NaOAc pH 5.5, 1 mM EDTA, 0.25% SDS), and the RNA precipitated with isopropanol. Samples were then 3’dephosphorylated by denaturing at 65 °C for 5 min and incubating with 1 μL 10× T4 PNK Buffer, 1 μL SUPERase Inhibitor, 1 μL 10 U/μL T4 PNK (NEB M0201S) in a 10 µL reaction at 37 °C for 1 h followed by heat inactivation at 65 °C for 20 min. Samples were then incubated with 0.5 μL of 50 μM Universal miRNA Cloning Linker (NEB S1315S), denatured at 65 °C for 5 min, and mixed with 1 μL T4 RNA Ligase 2, truncated KQ (NEB M0373S), 1 μL 10x buffer, 6 μL 50% PEG 8000, and 1.5 μL water for 4.5 h at 25 °C. The free adaptor was then removed by addition of 1 μL 10 U/μL 5’-Deadenylase (NEB M0331S), 1 μL 10 U/μL RecJ Exonuclease (Lucigen/Epicentre, RJ411250), and 1 μL 20 U/μL SUPERase Inhibitor and incubation at 30 °C for 1 h. Samples were then purified using Zymo RNA Clean and Concentrator-5 columns using the protocol above to preserve small RNAs (100 μL sample, 200 μL RNA binding buffer, 450 μL 100% ethanol). Each 10 μL sample was then incubated with 2 μL of 1.25 μM reverse transcription primers containing sample barcodes and unique molecular identifiers and denatured at 65 °C for 5 min, followed by reverse transcription by SuperScript III (Thermo Fisher, 18080-044) in a 20 μL reaction incubated at 25 °C for 5 min followed by 48 °C for 40 min. RNA was then hydrolyzed by adding 2.2 μL of 1 N NaOH and incubating for 20 min at 98 °C. Samples were then purified using Zymo RNA Clean & Concentrator-5 kit (100 μL sample, 200 μL RNA binding buffer, 450 μL 100% ethanol) and run on a 10% TBE-urea polyacrylamide gel. cDNA was gel extracted using DNA extraction buffer (300 mM NaCl, 10 mM Tris-HCl pH 8, 1 mM EDTA, 0.1% SDS) overnight and precipitated with isopropanol at −80 °C overnight. Samples were then circularized with CircLigase (Illumina, CL4115K) in a 20 μL reaction (15 μL cDNA, 2 μL 10x CircLigase Buffer, 1 μL 1 mM ATP, 1 μL MnCl_2_, 1 μL CircLigase) for 12 h at 60 °C followed by a 10-min heat inactivation at 80 °C. Samples were purified using Zymo RNA Clean & Concentrator-5 columns (100 μL sample, 200 μL RNA binding buffer, 450 μL 100% ethanol) and eluted with 12 μL 10 mM Tris-HCl pH 8. 1 μL of the library was used for PCR amplification with Phusion High-Fidelity DNA Polymerase (Thermo Fisher F530S) (98 °C 30 s, 98 °C 10 s, 65 °C 10 s, 72 °C 5 s) for ten cycles. PCR product was run on native 8% TBE polyacrylamide gels, extracted overnight using DNA extraction buffer, and isopropanol precipitated for at least 2 h at −80 °C. Sample concentration and quality was determined by running on the Agilent 2100 Bioanalyzer (High-Sensitivity DNA) by the Stanford Protein and Nucleic Acid (PAN) Facility both before and after library pooling. Libraries were sequenced by the Stanford Functional Genomics Facility (SFGF) on the Illumina NextSeq 500 (1x75nt). The sequences of all oligos used in sample preparation are included in Supplementary Table 1.

Ribosome profiling analysis was performed as previously described^26^. In brief, reads were demultiplexed, barcode and adaptor sequences removed, and quality filtered using UMI-tools (v0.5.4), FASTX-Toolkit (v0.0.13), and cutadapt (v1.14). Reads that aligned to rRNA, tRNA, or snRNA using bowtie2 were discarded; the remaining reads were aligned to GRCm38/mm10 transcriptome reference derived from UCSC/GENCODE VM20 knownCanonical annotations filtered for high confidence transcripts. PCR duplicates were then removed using UMI-tools. Ribosome A site positions were determined by offsetting the distance of the 5’ end of each ribosome-protected fragment (RPF) read to canonical start sites in each length group and adding 4 nucleotides. Reads aligning to the CDS (with the first 15 codons and last 5 codons removed) were used for RPF libraries, and reads aligning to the entire transcript were used for RNA-Seq libraries. Transcripts with counts per million (cpm) >0.75 for at least three ribosome profiling libraries were retained for downstream analysis. RPF and RNA-Seq libraries were then normalized separately by the method of the trimmed mean of M-values (TMM) using edgeR. Differential translation analysis was performed using voom and limma, sva was used to remove batch effects from the contrast matrix, and significance was calculated using the locfdr R packages. Multiple testing correction was performed using the Benjamini-Hochberg method. Gene set enrichment analysis was performed using Camera^30^ with mouse GO Biological Processes gene sets obtained from http://download.baderlab.org/EM_Genesets/current_release/Mouse/Entrezgene/. Gene sets were filtered such that all genes in gene sets have expression values in the dataset and to only include those with >10 and <500 genes. Multiple testing correction was performed using the Benjamini-Hochberg method. Enriched gene sets were visualized using Enrichment Map and Cytoscape^49^ (v3.8.2).

### Embryo sucrose-gradient RT-qPCR

E9.5 embryos were lysed in lysis buffer (20 mM Tris pH 7.5, 150 mM NaCl, 15 mM MgCl_2_, 1 mM DTT, 8% glycerol, 1% Triton X-100, 100 μg/ml cycloheximide, 20 U/ml Turbo DNAse (Thermo Scientific, AM2238), and Complete Protease Inhibitor EDTA-free (Sigma-Aldrich, 11836170001)) at 4 °C for 30 min with occasional vortexing, followed by sequential centrifugation at 1800g for 5 min at 4 °C and then 10,000×*g* for 5 min at 4 °C. The clarified cytoplasmic extract was loaded onto 25–50% sucrose gradients (20 mM Tris pH 7.5, 100 mM NaCl, 15 mM MgCl_2_, 100 μg/ml cycloheximide), made by sequentially freezing 50%, 43.75%, 37.5%, 31.25%, and 25% sucrose. Gradients were centrifuged in a Beckman SW60 rotor at 35,000 rpm for 2.5 h at 4 °C in a Beckman L8-80M ultracentrifuge and then fractionated on a Density Gradient Fraction System (Brandel, BR-188) using PeakChart software (v1.02) with continuous A_260_ measurements. Each fraction was spiked with 100 pg of in vitro transcribed luciferase RNA and extracted using acid phenol-chloroform, incubating for 5 min at 65 °C followed by centrifugation at 21,000 ×*g* for 10 min. The aqueous phase was mixed 1:1 with ethanol and RNA isolated using the RNA Clean and Concentrator-5 Kit (Zymo Research). The RNA was treated with TURBO DNase (Thermo Scientific) for 30 min at 37 °C and purified with the RNA Clean and Concentrator-5 Kit (Zymo Research). cDNA synthesis was performed using the iScript Supermix (Bio-rad) with 20 ng of RNA, diluted 20-fold, and 4 µl used as a template for qPCR with SsoAdvanced SYBR Green supermix (Bio-Rad) on a CFX384 machine (Bio-rad). All primers are listed in Supplementary Table 1. The Ct value of each mRNA of interest from each fraction was first normalized to the Ct value of the luciferase RNA spike-in, converted from log2 to linear values, and normalized to the total abundance of the mRNA across all fractions. Significance between genotypes was calculated using Student’s *t* tests.

### Ribosome immunoprecipitation and RT-qPCR

3xFLAG-RPL10A/uL1 or RPL22/eL22-3xFLAG hESCs were differentiated into sclerotome and treated with 100 µg/mL cycloheximide at 37 °C for 2 min prior to harvesting and cytoplasmic extraction, with 100 µg/mL cycloheximide included in all buffers. Cytoplasmic extract was performed as described above using cytoplasmic lysis buffer (20 mM Tris pH 7.5, 150 mM NaCl, 15 mM MgCl_2_, 100 µg/ml cycloheximide, 1 mM DTT, 0.5% Triton X-100, 0.1 mg/ml heparin, 8% glycerol, 20 U/ml TURBO DNase (Ambion, AM2238), 200 U/mL SUPERase In RNase Inhibitor (Ambion, AM2696), 1x Combined Protease and Phosphatase Inhibitor (Thermo Scientific, 78443)). When performing the immunoprecipitation with RNase treatment, RNase inhibitor was initially omitted from the cytoplasmic lysis buffer; the cytoplasmic extract RNA concentration was measured by Nanodrop; cytoplasmic extracts were treated with 0.5 μg RNase A (Thermo Scientific, AM2271) and 300 U RNase T1 (Thermo Scientific, EN0541) per 75 μg of RNA for 30 min at room temperature with gentle rocking; and then SUPERase In RNase Inhibitor (at a volume three times that of the combined RNase volume) was added to halt the reaction. In total, 300 µL of cytoplasmic extract was layered onto a sucrose cushion (1 M sucrose in 20 mM Tris pH 7.5, 15 mM MgCl_2_, 150 mM NaCl, 1 mM DTT, 100 µg/ml cycloheximide, 200 U/mL SUPERase In RNase Inhibitor (Ambion, AM2696), 1× combined protease and phosphatase inhibitor (Thermo Scientific, 78443)) and spun in a Beckman TLA 120.2 rotor at 70,000 rpm for 4 h at 4 °C in a Beckman TL-100 ultracentrifuge to pellet the ribosomes. The supernatant was transferred to a new tube, and the protein precipitated using the Proteoextract Protein Precipitation Kit (Calbiochem) following kit instructions. The ribosomal pellet was resuspended in cytoplasmic lysis buffer, and protein concentration was measured using the BCA protein assay kit (Thermo Scientific). Approximately 10% of the cushion pellet sample was saved as the protein input sample and 10% as the RNA input sample. The remaining cushion pellet volume was brought up to 500 µL using cytoplasmic lysis buffer and FLAG-tagged ribosomes were immunoprecipitated by incubating with anti-FLAG M2 affinity gel (Sigma A2220), using a ratio of 75 µL resin per 1 mg of protein, for 2 h at 4 °C on a head-to-tail rotator. Resin was pellet by spinning at 1000 ×*g* for 1 min at 4 °C and the flow-through was transferred to a new tube, and the protein was precipitated using the Proteoextract Protein Precipitation Kit (Calbiochem) following kit instructions. The resin was washed three times for 5 min each at 4 °C with wash buffer 1 (20 mM Tris-HCl pH 7.5, 150 mM NaCl, 15 mM MgCl_2_, 0.5% Triton X-100, 1 mM DTT, 100 µg/mL cycloheximide), with the first wash saved by transferring to a new tube and the protein precipitated using the Proteoextract Protein Precipitation Kit (Calbiochem) following kit instructions. The resin was then washed another three times for 5 min each at 4 °C with wash buffer 2 (20 mM Tris-HCl pH 7.5, 300 mM NaCl, 15 mM MgCl_2_, 0.5% Triton X-100, 1 mM DTT, 100 µg/mL cycloheximide), with the first of these washes saved by transferring to a new tube and the protein precipitated using the Proteoextract Protein Precipitation Kit (Calbiochem) following kit instructions. The sample was then eluted off the beads by incubation for 30 min at 4 °C in elution buffer (25 mM Tris-HCl pH 7.5, 150 mM NaCl, 0.2 mg/mL 3xFLAG peptide (Sigma F4799)) using 2x the volume of resin. Several microliters were saved as protein elution sample; the rest was used as the RNA elution sample. RNA was extracted from the elution and input samples using TRIzol (Thermo Fisher) and used for cDNA synthesis using iScript Supermix (Bio-rad), following the manufacturer’s instructions. qPCR performed with SsoAdvanced SYBR Green supermix (Bio-Rad, catalog no. 1725270) on a CFX384 machine (Bio-rad). All primers are listed in Supplementary Table 1. The amount of each target and control mRNA was normalized to the abundance of *β-actin*, and then the amount of each mRNA in the elution was normalized to the amount in the input. Significance was calculated using Student’s *t* test.

### X-gal staining

E8.5–E10.5 mouse embryos were fixed in 0.2% glutaraldehyde, 5 mM EDTA, 2 mM MgCl_2_ in PBS for 2 h at 4 °C. Embryos were washed with 2 mM MgCl_2_, 0.2% NP-40 in PBS twice for 15 min each on an orbital shaker at room temperature and then incubated with staining solution (5 mM potassium ferricyanide, 5 mM potassium ferrocyanide, 2 mM MgCl_2_, 1 mg/mL X-gal in PBS) at 4 °C. Once the desired staining intensity has been achieved, the embryos were washed with PBS three times and stored in PBS + 0.1% Tween-20. Homozygous mutant embryos and littermate controls were processed and imaged in parallel. Quantification was performed using ImageJ. A common signal intensity threshold across all genotypes was set, and a region of interest (ROI) set around the target tissue. The area of signal above the threshold within the ROI was calculated relative to the total area of the ROI. For E8.5 embryos, images of the dorsal side of the embryos were used, and the ROI was set as the region posterior to the inflection point of the embryo. For E9–E10.5 embryos, lateral images were used, and the tail bud ROI was set from the distal tip of the tail bud to the position of the most posterior somite. The forelimb bud was also set as an ROI for E9.5–E10.5 embryos, and the neural tube from the level of the otic placode to the most posterior somite for E10.5 embryos.

### Trachea transmission electron microscopy

E18.5 embryo tracheas were isolated with the larynx still attached to identify the expected direction of polarity and prepared for transmission electron microscopy following established protocols^50^. Sectioning and imaging were performed by the Stanford Cell Sciences Imaging Facility. The angle of each basal foot relative to the orientation of the trachea was measured using ImageJ, with the proximal direction (toward the larynx) set to 0°. Data visualization and statistical analyses were performed using the Oriana software package (Kovach Computing Services) and the Circular package in R.

### Statistics and reproducibility

All measurements are made from individual biological replicates, and no data were excluded from the analyses. Mouse embryo experiments were each performed on multiple embryos harvested from multiple litters. No statistical methods were used to predetermine the sample size. The investigators were not blinded to allocation during experiments and outcome assessment. All statistical tests employed were two-sided. Unless otherwise stated in the figure legends, significance was calculated using two-tailed Student’s *t* tests with unequal variance.

### Reporting summary

Further information on research design is available in the Nature Research Reporting Summary linked to this article.

## Supplementary information

Supplementary Information

Description of Additional Supplementary Files

Supplementary Data 1

Supplementary Data 2

Supplementary Data 3

Supplementary Movie 1

Reporting Summary

## Data Availability

All processed mass spectrometry and sequencing data are available in the supplementary data files. Raw sequencing data have been deposited in the Gene Expression Omnibus under accession number GSE177520. Raw mass spectrometry data have been deposited in the ProteomeXchange with identifier PXD032904. The structure of the human ribosome utilized here was accessed from the Protein Data Bank (PDB: 4v6x). Materials may be obtained from the corresponding author upon request. Source data are provided with this paper.

## Source data

Source Data
