# Supplementary material for: A stem cell roadmap of ribosome heterogeneity reveals a function for RPL10A in mesoderm production
Source: Nat Commun. 2022 Sep 19;13:5491. doi: 10.1038/s41467-022-33263-3 (PMC9485161; doi:10.1038/s41467-022-33263-3)
Supplement: Supplementary file 1 — Supplementary Information [file 41467_2022_33263_MOESM1_ESM.pdf]

A stem cell roadmap of ribosome heterogeneity reveals a function for RPL10A in mesoderm production

Naomi R. Genuth, Zhen Shi, Koshi Kunimoto, Victoria Hung, Adele F. Xu, Craig H. Kerr, Gerald C. Tiu, Juan A. Oses-Prieto, Rachel E.A. Salomon-Shulman, Jeffrey D. Axelrod, Alma L. Burlingame, Kyle M. Loh, Maria Barna

<u>Supplementary Information</u>

Supplementary Figures 1-16

Supplementary Table 1

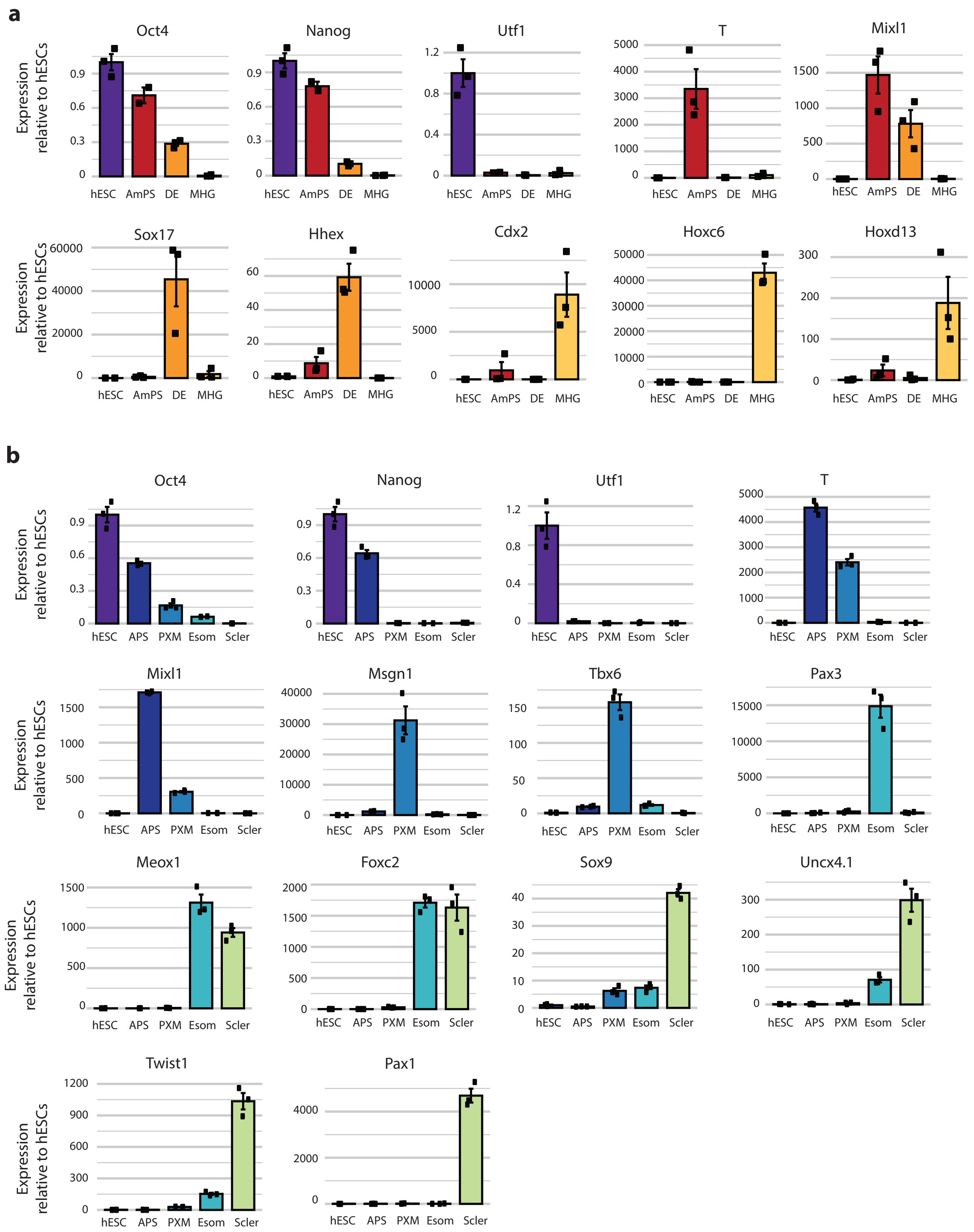

Supplementary Figure 1. hESC in vitro differentiation marker gene expression. Analysis of marker gene expression by qPCR for the endoderm **(a)** and mesoderm **(b)** lineages (n=3 for each cell type). Colors match the colors of the cell differentiation schematic in Figure 1. Values for each gene were normalized to the expression of a housekeeping gene (*Nupl1*) and presented as mean +/- SEM. All genes show the expected enrichments in the specific cell types. Source data are provided as a Source Data file. AmPS=anterior-most primitive streak; DE=definitive endoderm; MHG=mid/hindgut; APS=anterior primitive streak; PXM=paraxial mesoderm; ESom=early somite; Scler=sclerotome.

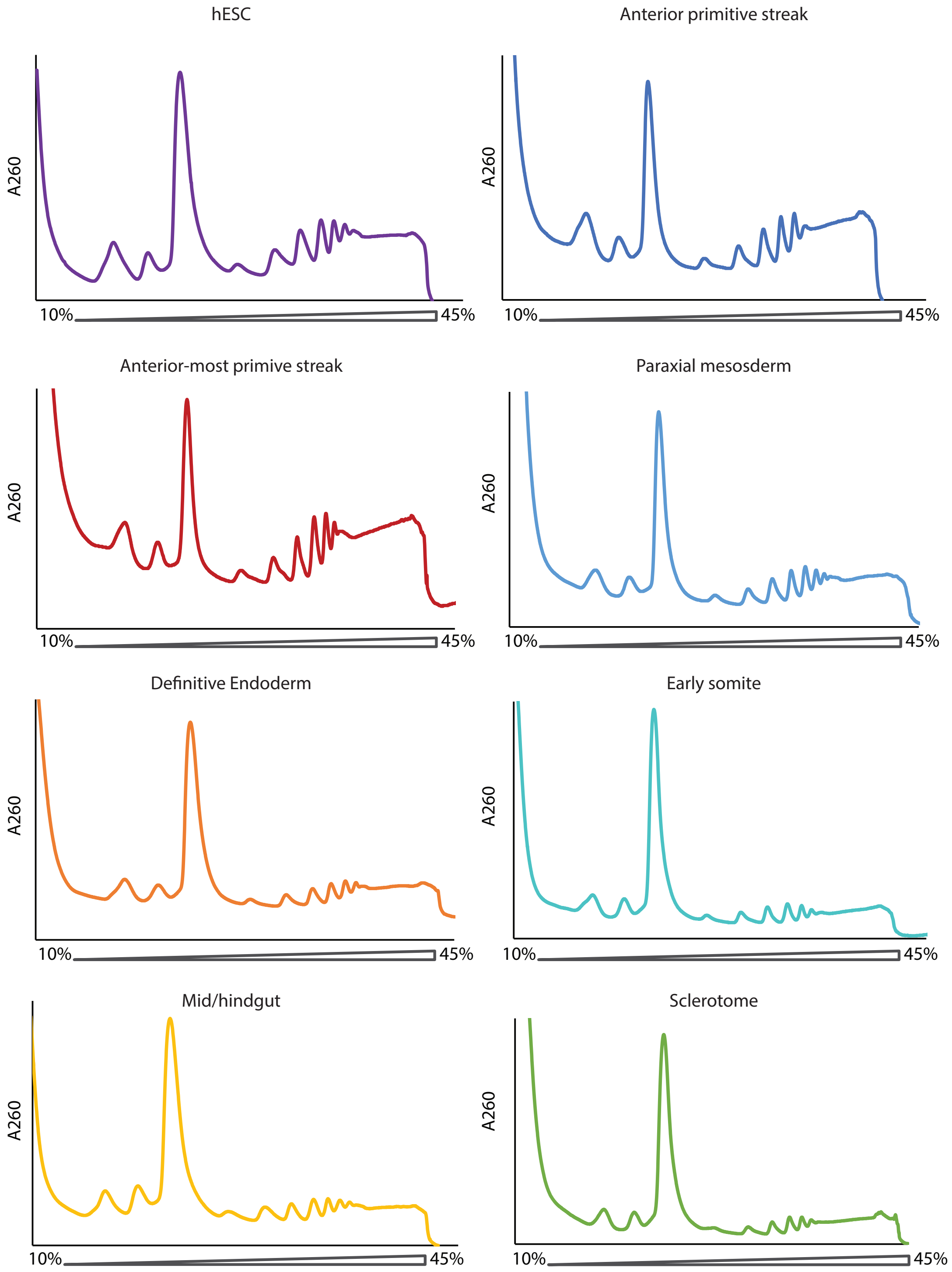

Supplementary Figure 2. hESC in vitro differentiation sucrose gradient fractionation. Polysome traces from 10-45% sucrose gradients of hESCs and each differentiated cell type used for ribosome mass spectrometry. Colors match cell differentiation schematic in Figure 1.

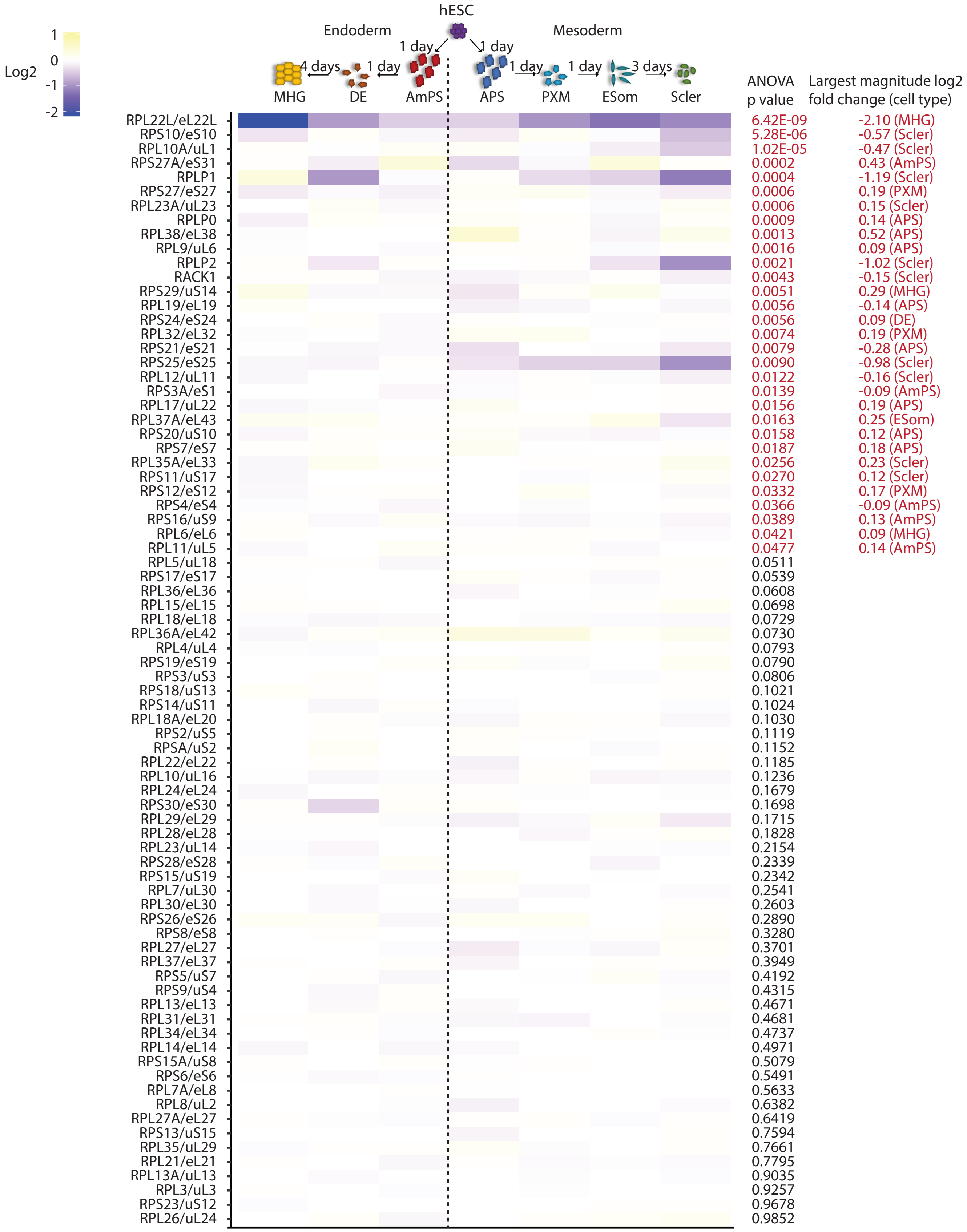

Supplementary Figure 3. Relative quantification mass spectrometry of polysome ribosome composition during hESC differentiation. Complete heat map of polysome relative abundance TMT mass spectrometry results. Values are the median ratios of polysome abundance in each differentiated cell relative to hESCs in log2 scale, n=6 for each differentiated cell type except for APS and MHG (n=7 each), and p values were calculated by ANOVA. The p values less than 0.05 are marked in red. For each significantly changing RP, the cell type where the RP shows the greatest magnitude of change is listed, along with the relative polysomal abundance of that RP in that cell type (values in log2 scale). AmPS=anterior-most primitive streak; DE=definitive endoderm; MHG=mid/hindgut; APS=anterior primitive streak; PXM=paraxial mesoderm; ESom=early somite; Scler=sclerotome.

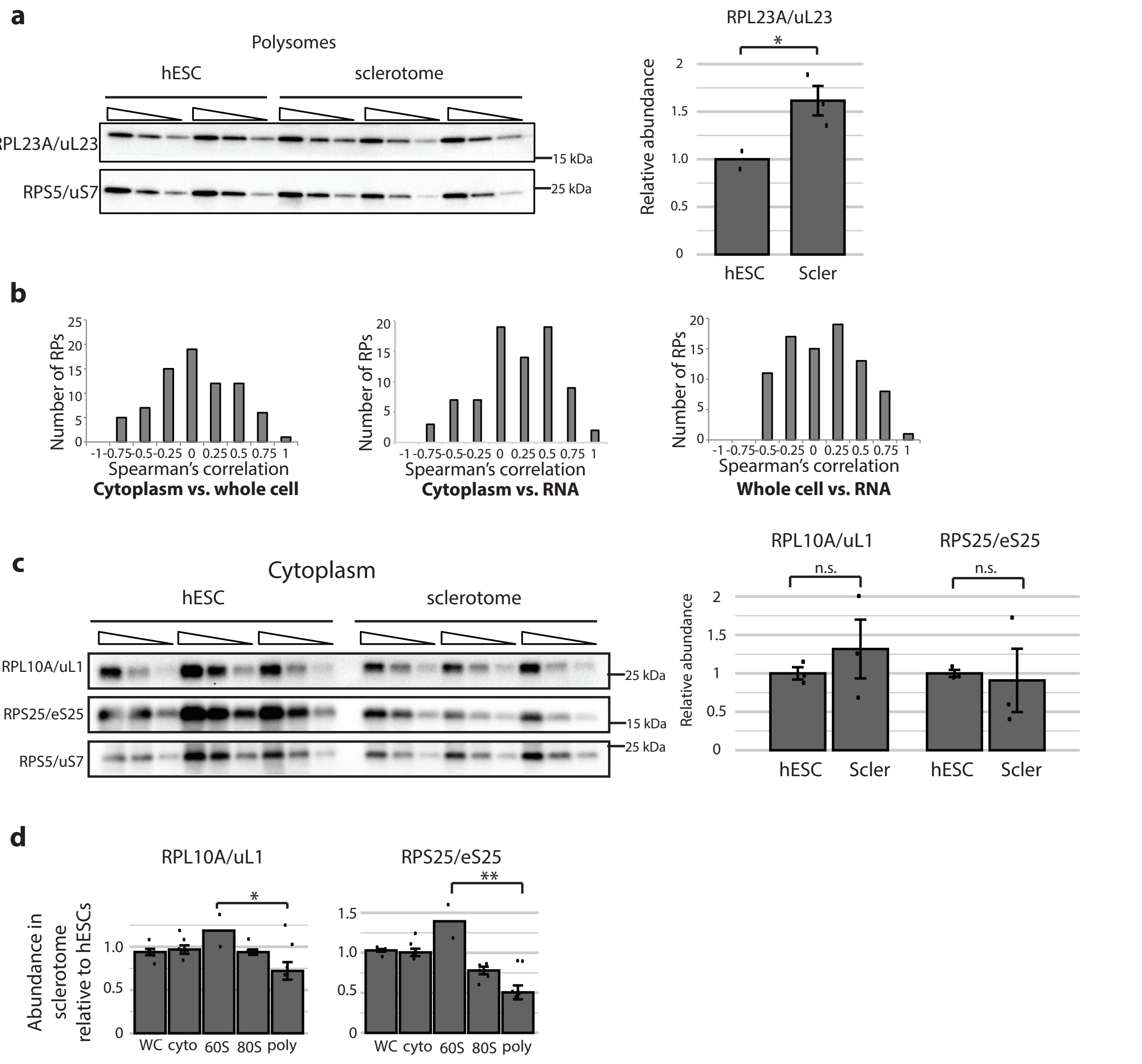

Supplementary Figure 4. Regulation of ribosome composition at multiple levels. (a) Western blot of RPs in hESC (n=2) and sclerotome (n=3) polysome samples. Each sample was loaded as a serial dilution (1, 0.5, 0.25 µg) and RPL23A/uL23 abundance was quantified relative to the non-heterogeneous RPS5/uS7. Values graphed are average quantification of the middle dilution (0.5 µg) with SEM error bars, and significance was calculated using Student's t tests (p=0.046). **(b)** Histograms of Spearman's correlation coefficients for each RP comparing cytoplasmic (n=6 for all cell types except for n=8 for mid/hindgut samples) and whole-cell protein abundance (n=5 for early somite, paraxial mesoderm, and mid/hindgut; n=6 for anterior primitive streak, sclerotome, anterior-most primitive streak, and definitive endoderm), cytoplasmic protein and mRNA abundance (n=3 for mesoderm lineage, n=1 for endoderm lineage), and whole-cell protein and mRNA abundance. **(c)** Western blot of RPs in hESC (n=3) and sclerotome (n=3) cytoplasmic extract samples. Each sample was loaded as a serial dilution (10, 5, 2.5 µg) and RPL10A/uL1 and RPS25/eS25 abundance was quantified relative to the non-heterogeneous RPS5/uS7. Values graphed are average quantification of the middle dilution (5 µg) with SEM error bars, and significance was calculated using Student's t test. **(d)** Relative abundance of RPL10A/uL1 and RPS25/eS25 in the sclerotome polysomes (poly) (n=6), monosomes (80S) (n=5), free subunits (40S or 60S) (n=2), cytoplasmic extract (cyto) (n=6), and whole cell extract (WC) (n=6) relative to hESCs. Values are median ratios with standard error bars. Significance between polysomal and free subunit abundance was calculated by ANOVA (RPL10A/uL1 p=0.01, RPS25/eS25 $p=3*10^{-6}$). * = $p < 0.05$; ** = $p < 0.01$. Source data are provided as a Source Data file.

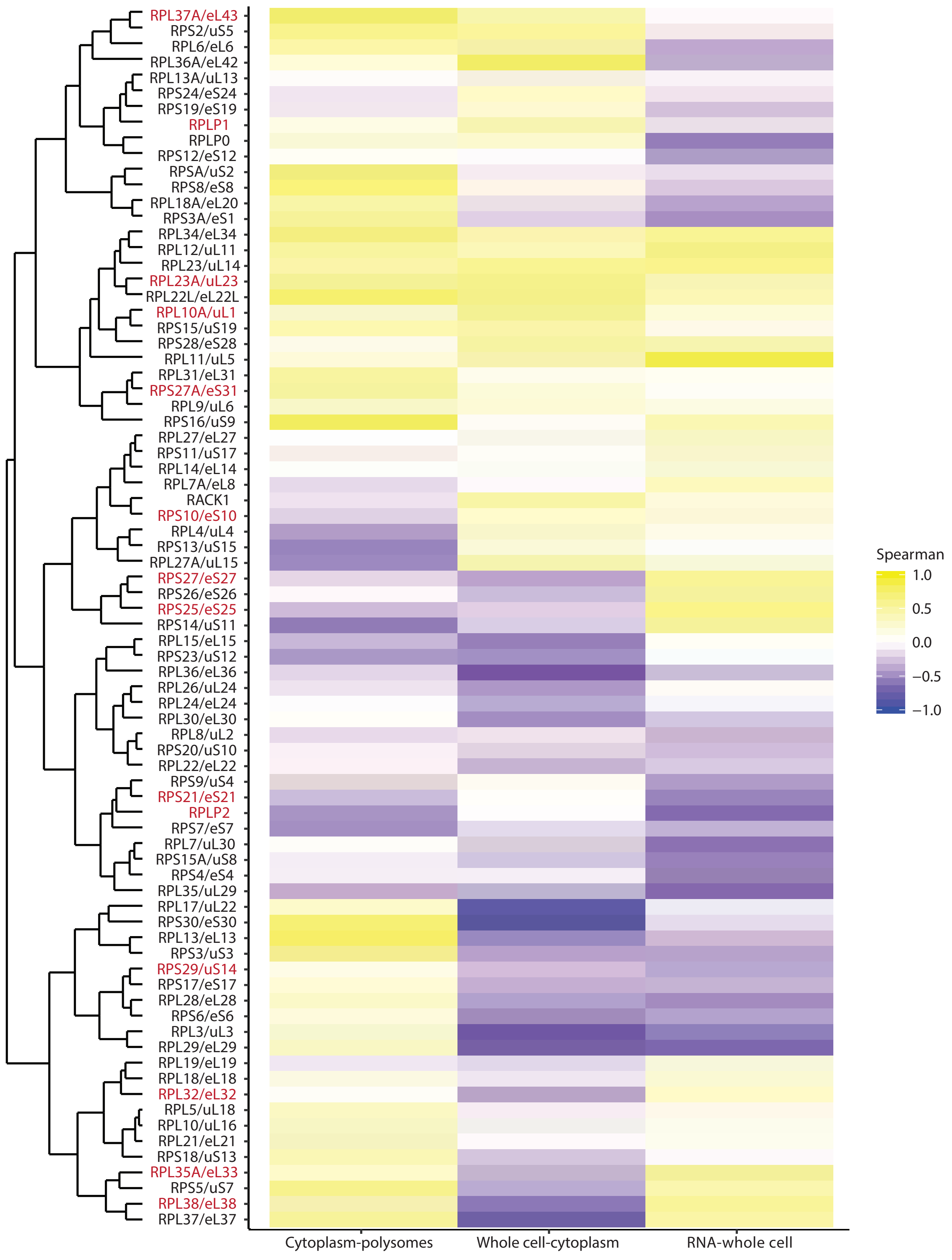

Supplementary Figure 5. Correlation of RP abundance changes across cellular compartments. Heatmap of Spearman's correlation coefficients for each RP comparing mRNA and whole-cell protein abundance, whole-cell and cytoplasmic protein abundance, and cytoplasmic and polysomal protein abundance. The RPs that are depicted in Figure 1C (changed significantly in the polysomes and change by at least 10% in at least 2 differentiated cell types relative to hESCs) are marked in red.

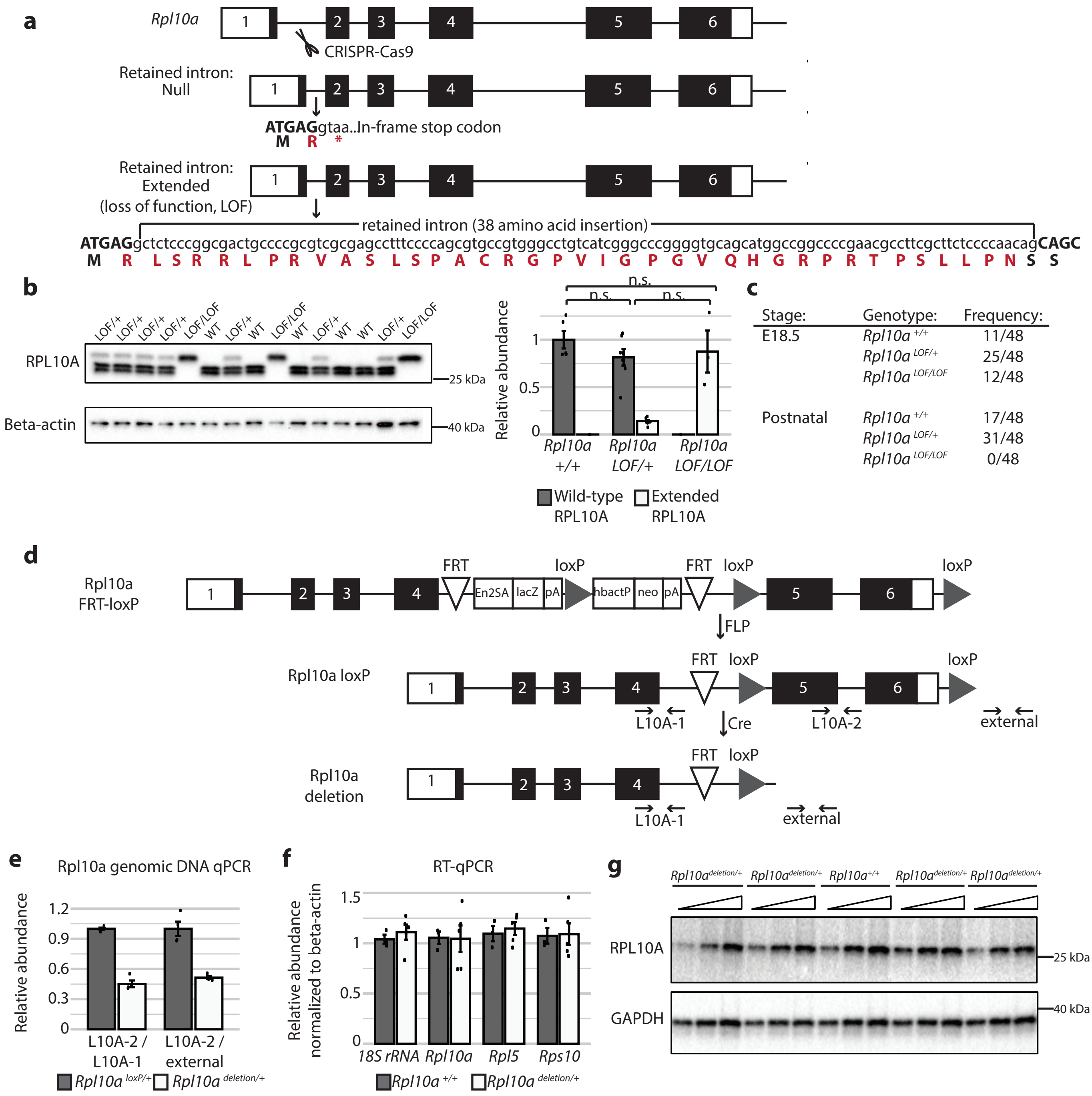

Supplementary Figure 6. Creation of *Rpl10a* mouse models. (a) Schematics of the wild-type *Rpl10a* mouse locus, the extended and null alleles created by CRISPR-mediated deletion of part of the intron between exons 1 and 2. **(b)** Western blot of RPL10A/uL1 and β-actin in wild-type, *Rpl10a* $^{LOF/+}$, and *Rpl10a* $^{LOF/LOF}$ embryo E9.5 whole cell lysates. Abundance of wild-type and extended RPL10A/uL1 protein was quantified for each genotype (n=5 for *Rpl10a*$^{+/+}$, n=7 for *Rpl10a* $^{LOF/+}$, n=3 for *Rpl10a* $^{LOF/LOF}$) and graphed as the average with SEM error bars, and significance was calculated using Student's t test. **(c)** Frequency of recovery of wild-type, *Rpl10a* $^{LOF/+}$, and *Rpl10a* $^{LOF/LOF}$ embryos from crossing *Rpl10a* $^{LOF/+}$ heterozygotes. At E18.5 the genotypes are present at the expected Mendelian ratios; postnatally (P1-P7), no *Rpl10a* $^{LOF/LOF}$ mice were recovered. **(d)** Schematic of the conditional allele used to generate an *Rpl10a* deletion allele. **(e)** Genomic DNA qPCR to confirm Cre excision of *Rpl10a*. SEM error bars, n=3 each for *Rpl10a* $^{loxp/+}$ and *Rpl10a* $^{deletion/+}$. **(f)** Reverse transcription qPCR of *Rpl10a* mRNA and controls to evaluate *Rpl10a* expression. Values are normalized by the abundance of *β-actin* mRNA. SEM error bars, n=3 for control and n=5 for *Rpl10a* $^{deletion/+}$. **(g)** Western blot to detect RPL10A/uL1 and GAPDH in wild-type and *Rpl10a* $^{deletion/+}$ E10.5 embryos. Source data are provided as a Source Data file.

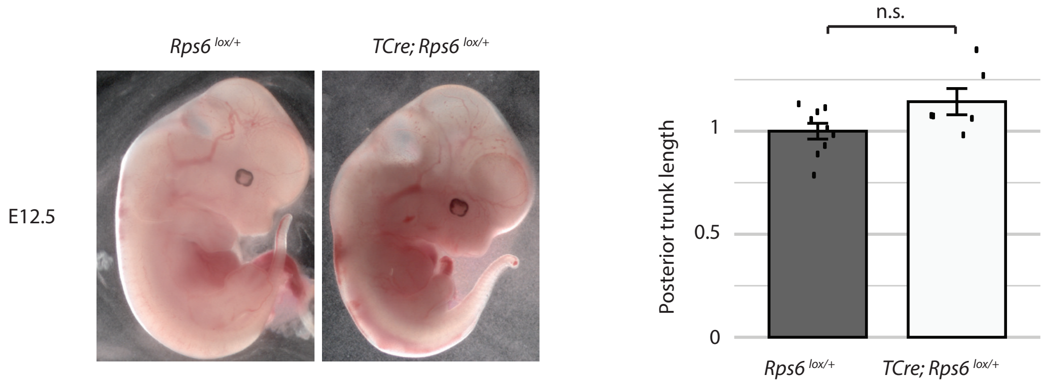

Supplementary Figure 7. Posterior trunk length is unaffected in *RPS6* haploinsufficient mice. Lateral views of E12.5 *RPS6* $^{lox/+}$ and *T Cre*; *RPS6* $^{lox/+}$ embryos. Quantification of posterior trunk length indicates that *RPS6* conditional heterozygosity does not cause posterior trunk truncations. Values graphed are average trunk length with SEM error bars, and significance was calculated using Student's t tests. *RPS6* $^{lox/+}$ embryos n=9, and *T Cre*; *RPS6* $^{lox/+}$ embryos n=6. Source data are provided as a Source Data file.

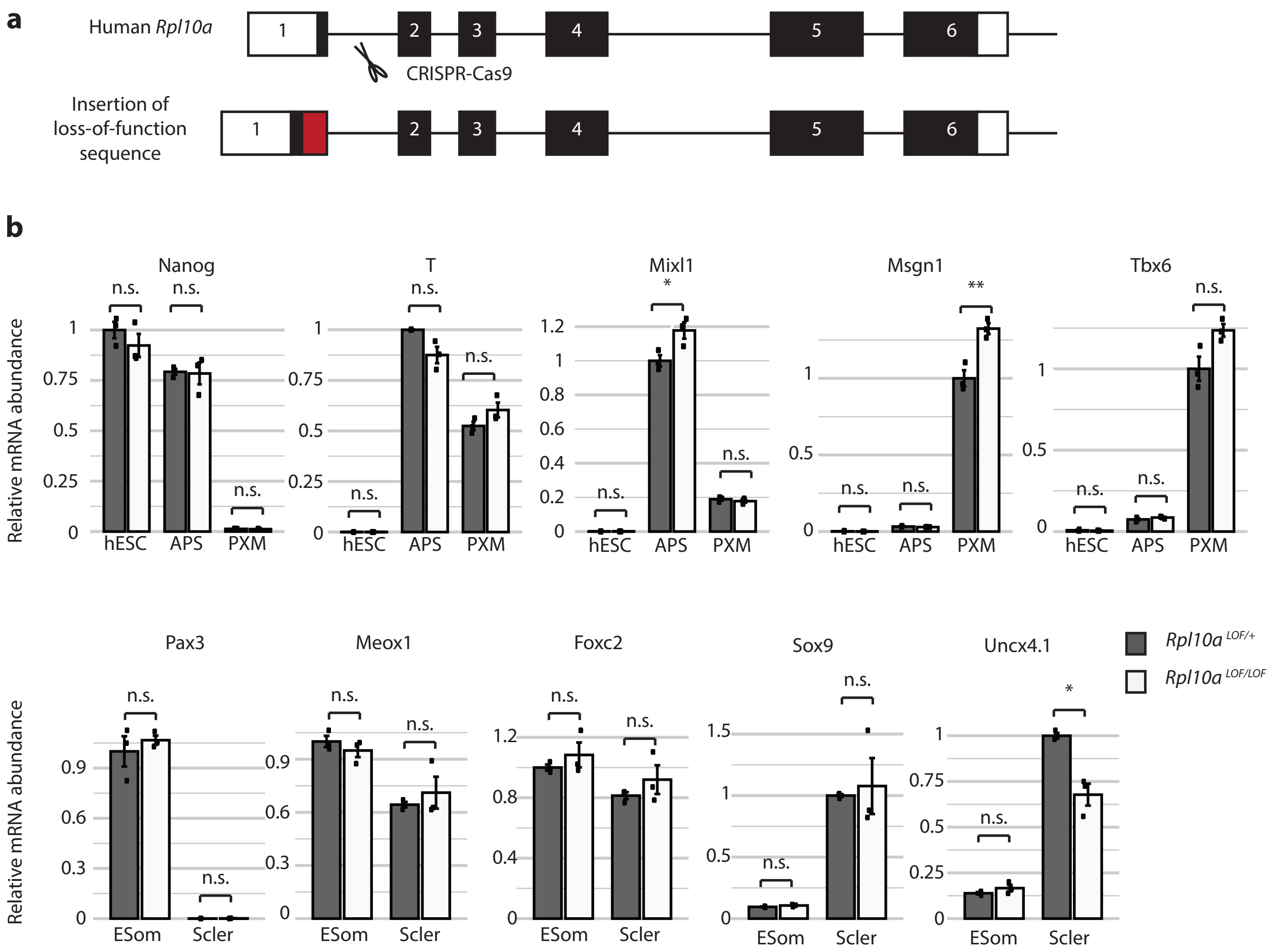

Supplementary Figure 8. Creation of *Rpl10a* loss-of-function hESC line. (a) Schematic of CRISPR to introduce LOF insertion into the *Rpl10a* locus in hESCs. **(b)** RT-qPCR of lineage markers in *Rpl10a* $^{LOF/+}$ and *Rpl10a* $^{LOF/LOF}$ hESCs and *in vitro* differentiated anterior primitive streak (APS), paraxial mesoderm (PXM), early somites (ESom), and sclerotome (Scler) (n=3 for each cell type). Values are normalized to control gene *Nupl1* and the average with SEM error bars shown, and significance was calculated using Student's t test. P values: APS *Mixl1* p=0.04; PXM *Msgn1* p=0.009; Scler *Uncx4.1* p=0.03. * = p value < 0.05; ** = p value < 0.01. Source data are provided as a Source Data file.

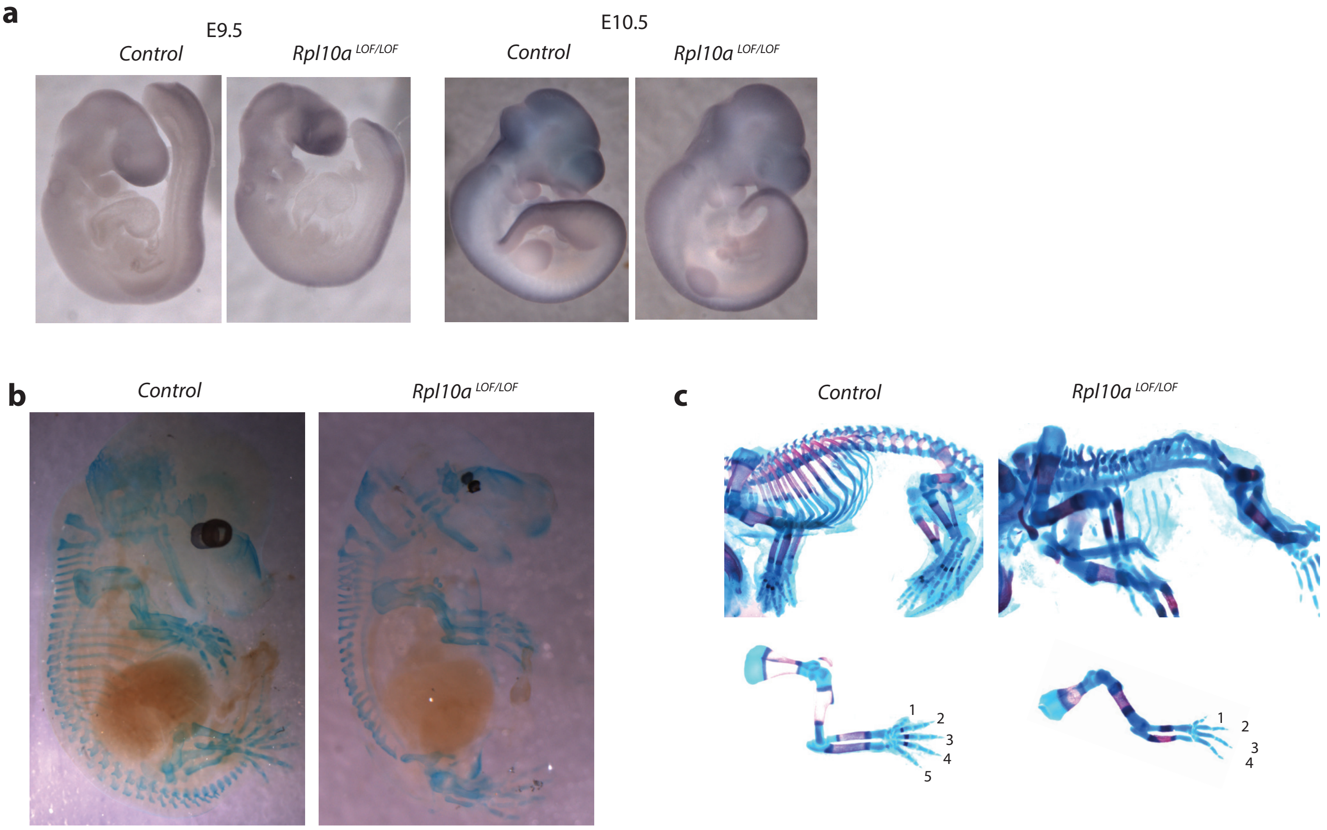

Supplementary Figure 9. *Rpl10a* $^{LOF/LOF}$ embryo neural tube, cartilage, and bone formation. **(a)** Whole-mount *in situ* hybridizations in control and *Rpl10a* $^{LOF/LOF}$ embryos at E9.5 and E10.5 for *Sox2*. **(b)** Cartilage staining (blue) on E14.5 *Rpl10a* $^{LOF/LOF}$ and control embryos. **(c)** Bone (red) and cartilage (blue) staining on E17.5 *Rpl10a* $^{LOF/LOF}$ and control embryos.

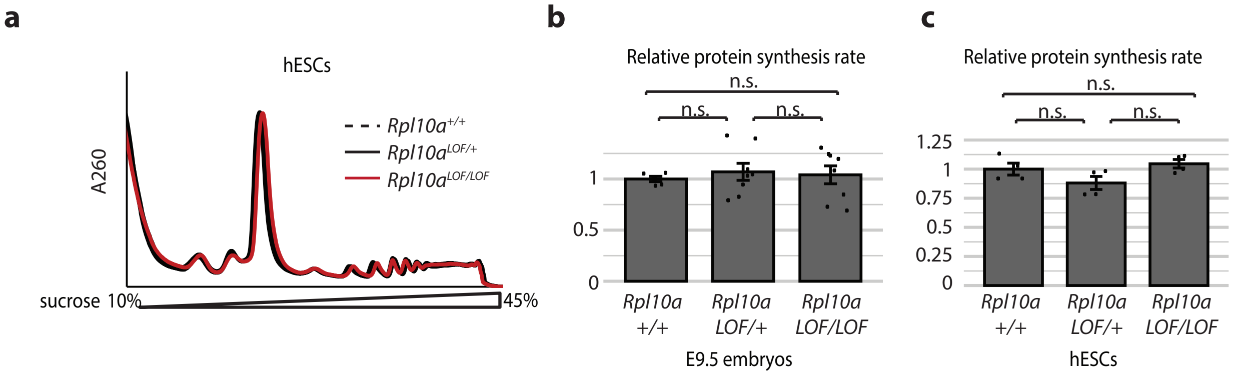

Supplementary Figure 10. Global protein synthesis is unchanged in *Rpl10a* $^{LOF/LOF}$ embryos and hESCs. (a) Overlay of polysome traces of 10-45% sucrose gradients of wild-type, *Rpl10a* $^{LOF/+}$, and *Rpl10a* $^{LOF/LOF}$ hESCs. **(b)** OPP incorporation rates in E9.5 wild-type, *Rpl10a* $^{LOF/+}$, and *Rpl10a* $^{LOF/LOF}$ embryos (n=5 for wild-type, n=8 each for *Rpl10a* $^{LOF/+}$ and *Rpl10a* $^{LOF/LOF}$). Values graphed are averages with SEM error bars and significance was calculated using Student's t test. **(c)** OPP incorporation rates in wild-type, *Rpl10a* $^{LOF/+}$, and *Rpl10a* $^{LOF/LOF}$ hESCs (n=4 each). Values graphed are averages with SEM error bars and significance was calculated using Student's t test. Source data are provided as a Source Data file.

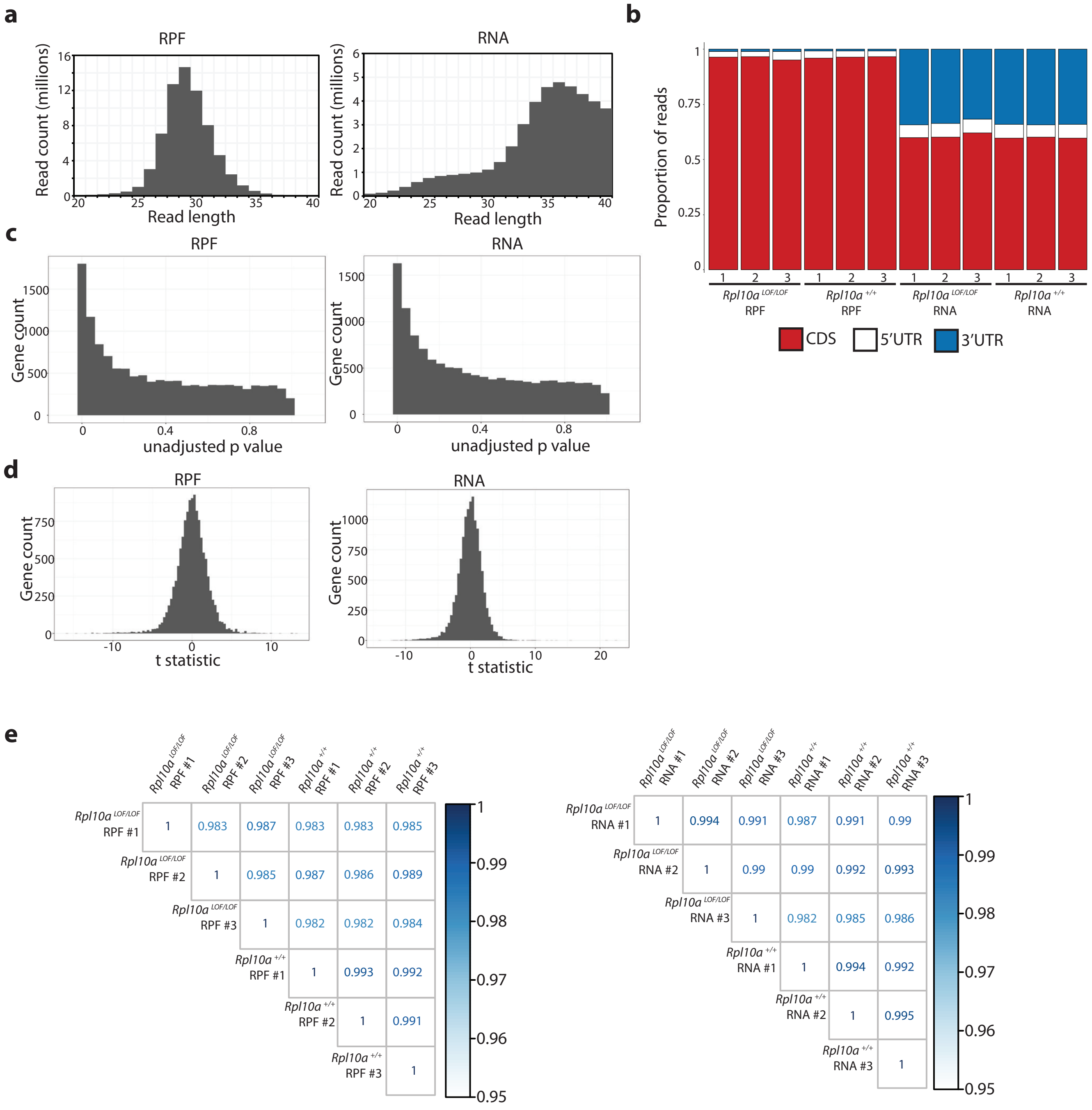

Supplementary Figure 11. *Rpl10a* $^{LOF/LOF}$ embryo ribosome profiling analysis. (a) Histogram of read lengths in ribosome profiling (RPF) and RNA-seq (RNA) libraries. **(b)** Distribution of reads across the 5' untranslated region (5'UTR), coding sequence (CDS), and 3' untranslated region (3'UTR) in each wild-type and *Rpl10a* $^{LOF/LOF}$ RNA-seq (RNA) and ribosome profiling (RPF) replicate. **(c)** Histogram of unadjusted p values for ribosome profiling (RPF) and RNA-seq (RNA) libraries. **(d)** Histogram of t statistics for ribosome profiling (RPF) and RNA-seq (RNA) libraries. **(e)** Correlation plots of each wild-type and *Rpl10a* $^{LOF/LOF}$ ribosome profiling (RPF) and RNA-seq (RNA) replicate.

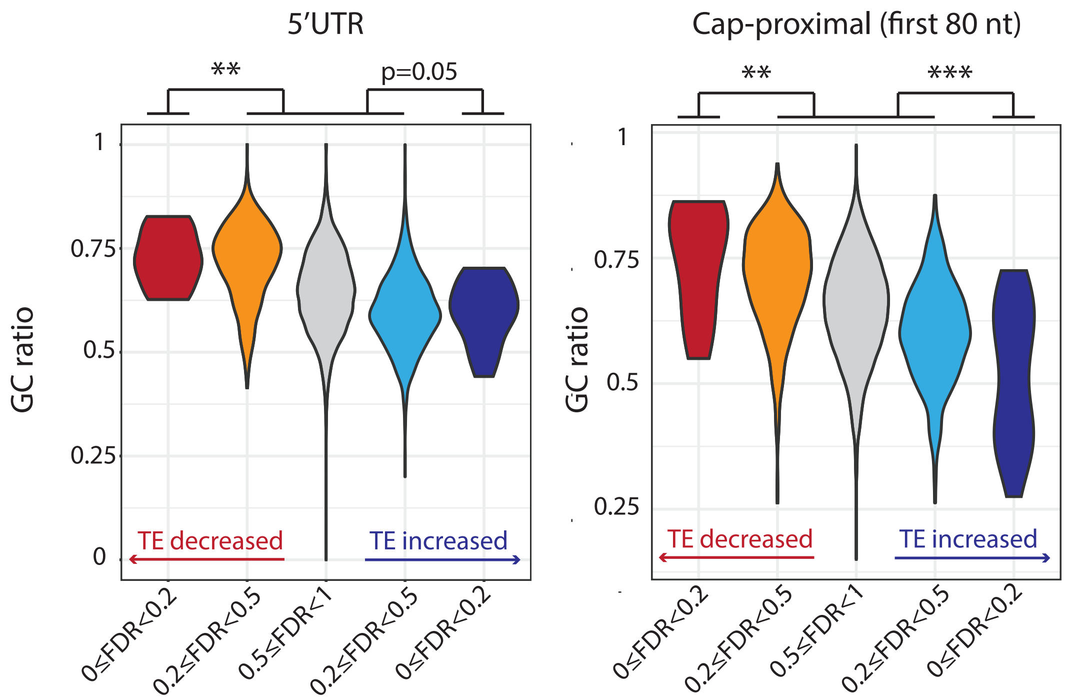

Supplementary Figure 12. Transcript features associated with altered translation in *Rpl10a* $^{LOF/LOF}$ embryos. Violin plots quantifying GC content of 5'UTRs (left) or cap-proximal regions (first 80 nucleotides, right). Transcripts are grouped by direction of change in translation efficiency (decreased translation in *Rpl10a* $^{LOF/LOF}$ embryos in red or orange, no change in gray, increased translation in blue) and by FDR. Significance was calculated using the Mann-Whitney test. P values: 5'UTR, TE decreased p=0.008; cap-proximal, TE decreased p=0.009; cap-proximal, TE increased $p=1.7*10^{-5}$. ** = p value < 0.01; *** = p value < 0.001.

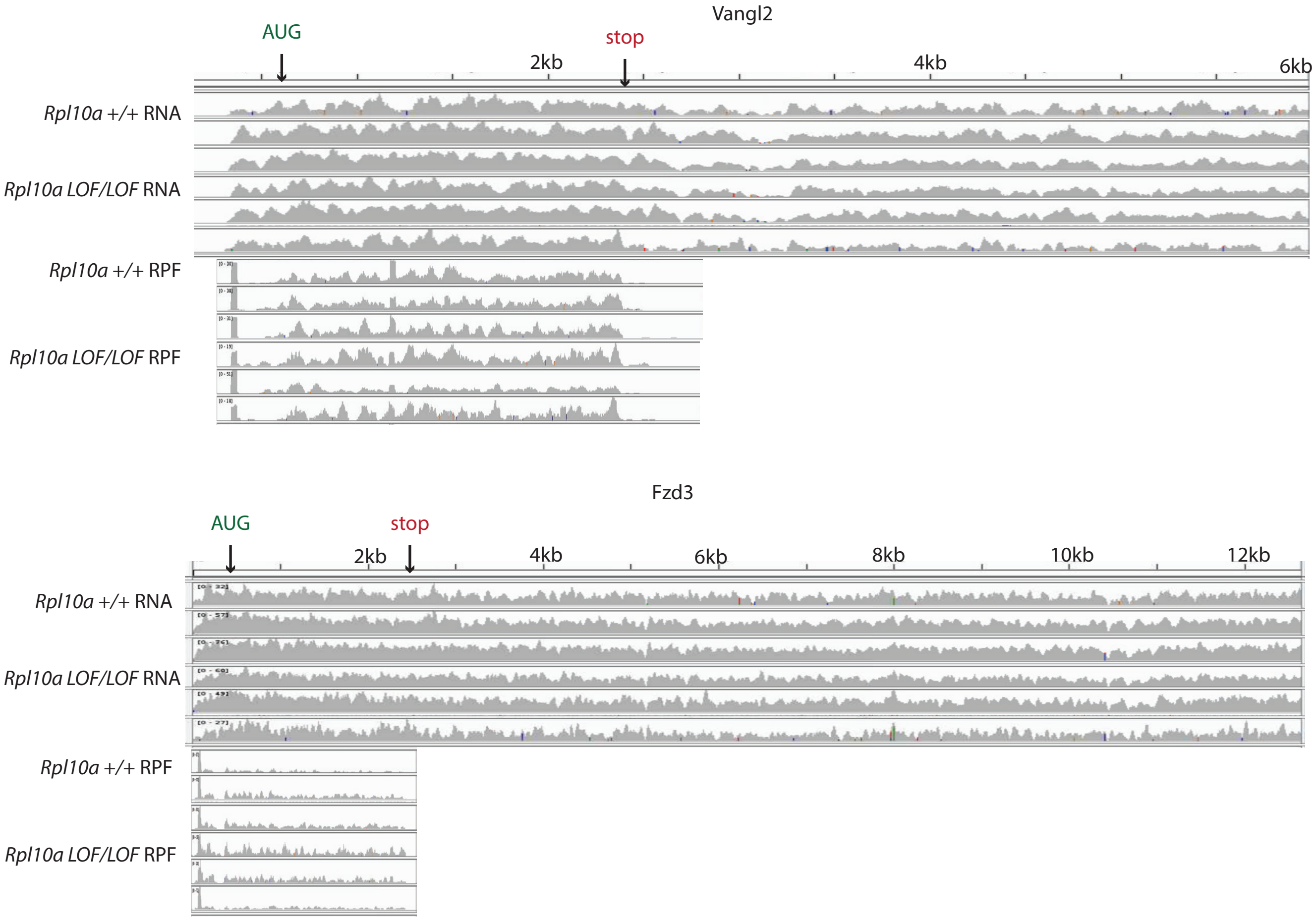

Supplementary Figure 13. Coverage of *Vangl2* and *Fzd3* in *Rpl10a* $^{LOF/LOF}$ embryo ribosome profiling. IGV views showing coverage of the *Vangl2* and *Fzd3* loci in each RNA-Seq and ribosome profiling replicate. Start and stop codons are marked as AUG and stop respectively.

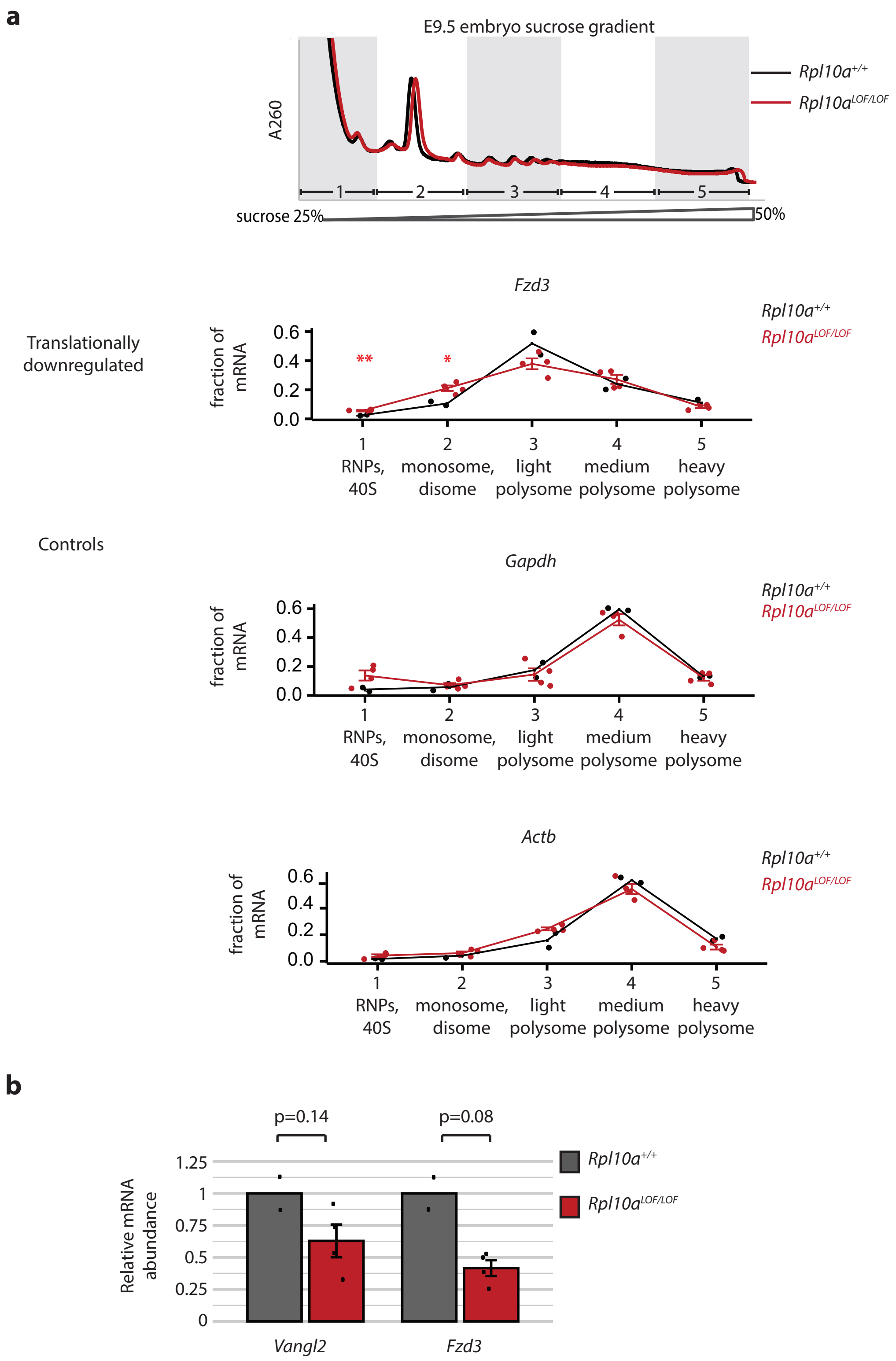

Supplementary Figure 14. *Rpl10a* $^{LOF/LOF}$ **embryo gradient qPCR reveals decreased translation of *Fzd3*. (a)** E9.5 wild-type (n=2) and *Rpl10a* $^{LOF/LOF}$ (n=4) polysome gradient RT-qPCR results for the translationally downregulated gene *Fzd3* and the control genes *Gapdh* and *Actb*. *Fzd3* is significantly increased in the *Rpl10a* $^{LOF/LOF}$ earlier fractions relative to wild-type (Student's t test p value = 0.007 for fraction 1, p=0.01 for fraction 2). Error bars are SEM. **(b)** Abundance of *Vangl2* and *Fzd3* mRNAs relative to *Actb* and *Gapdh* in the E9.5 cytoplasmic extract inputs for polysome gradients. Wild-type (n=2) and *Rpl10a* $^{LOF/LOF}$ (n=4), error bars are SEM, significance calculated using Student's t test. * = p value < 0.05; ** = p value < 0.01. Source data are provided as a Source Data file.

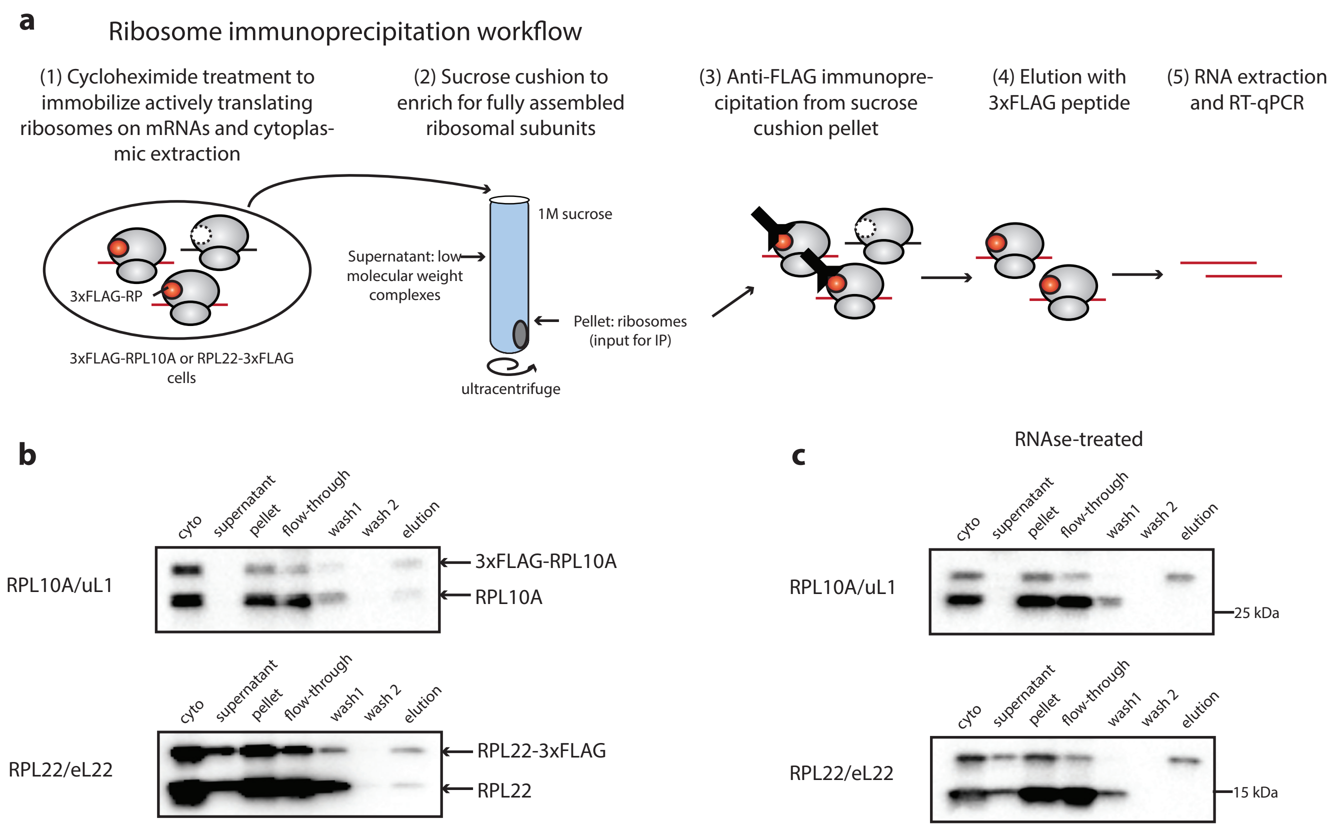

Supplementary Figure 15. 3xFLAG-RPL10A/uL1 and RPL22/eL22-3xFLAG ribosome immunoprecipitation. **(a)** Schematic of ribosome immunoprecipitation workflow. **(b)** Western of immunoprecipitation of 3xFLAG-RPL10A/uL1 or RPL22/eL22-3xFLAG ribosomes loading 2% of the volume of each sample. The untagged RPL10A/uL1 and RPL22/eL22 that appears in the elutions are due to untagged and tagged ribosomes forming a polysome together bound to the same mRNA molecule. Equivalent results were obtained from multiple independent experiments (n=4 each for 3xFLAG-RPL10A/uL1 and RPL22/eL22-3xFLAG) **(c)** Western of immunoprecipitation of 3xFLAG-RPL10A/uL1 or RPL22/eL22-3xFLAG ribosomes after treatment with RNAse A/T1 to cut unprotected mRNA, thereby cleaving polysomes into single monosomes. 2% of the volume of each sample was loaded. No untagged RPL10A/uL1 or RPL22/eL22 appears in these elutions because under these conditions only one ribosome can be present on a single mRNA. Equivalent results were obtained from multiple independent experiments (n=2 each for 3xFLAG-RPL10A/uL1 and RPL22/eL22-3xFLAG). Source data are provided as a Source Data file.

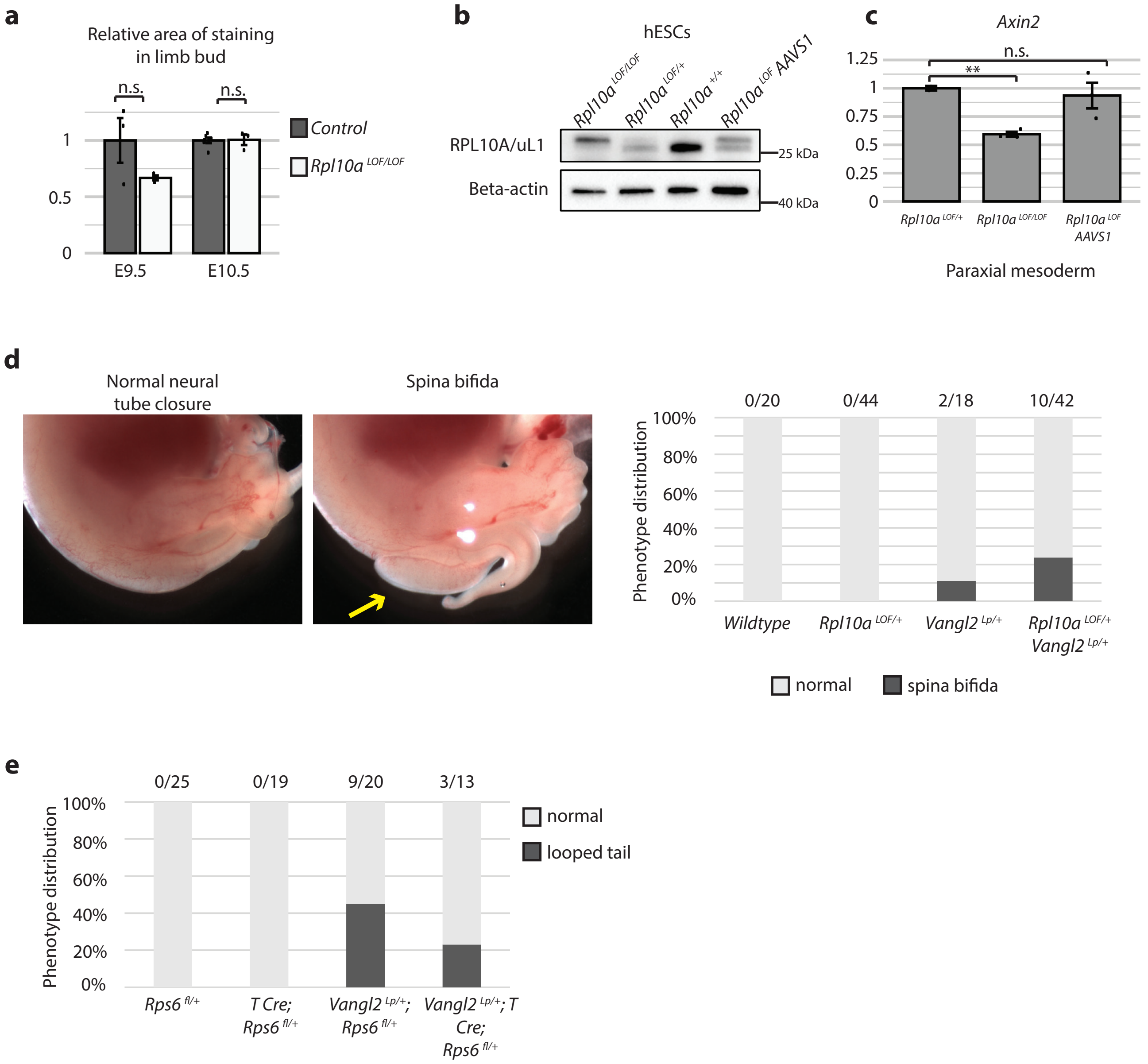

Supplementary Figure 16. Decreased canonical and non-canonical Wnt signaling in *Rpl10a* *LOF/LOF* embryos and *in vitro* differentiated paraxial mesoderm. (a) Quantification of area of X-gal staining in limb bud relative to overall limb bud area. E9.5 n=4, E10.5 n=3 for *Rpl10a* $^{LOF/LOF}$ and n=6 for control. Values shown are averages with SEM error bars, significance was calculated using Student's t test. **(b)** Western blot of RPL10A/uL1 and β-actin in whole cell lysates from wild-type hESCs, *Rpl10a* $^{LOF/+}$ hESCs, *Rpl10a* $^{LOF/LOF}$ hESCs, and hESCs with a *LOF-Rpl10a* transgene at the *AAVS1* locus (LOF AAVS1). **(c)** Abundance of *Axin2* mRNA relative to *Pbgd* control transcript as measured by RT-qPCR in *in vitro* differentiated paraxial mesoderm derived from *Rpl10a* $^{LOF/+}$ hESCs, *Rpl10a* $^{LOF/LOF}$ hESCs, and hESCs with a *LOF-Rpl10a* transgene at the *AAVS1* locus (LOF AAVS1) (n=3 each for *Rpl10a* $^{LOF/LOF}$ and *LOF-Rpl10a AAVS1*, n=2 for *Rpl10a* $^{LOF/+}$). Values shown are averages with SEM error bars and significance was calculated using Student's t test (p values 0.001 (*Rpl10a* $^{LOF/LOF}$), 0.63 (*LOF-Rpl10a AAVS1*)). **(d)** Frequency of spina bifida in wild-type, *Rpl10a* $^{LOF/+}$, *Vangl2* $^{Lp/+}$, and *Rpl10a* $^{LOF/+}$ *Vangl2* $^{Lp/+}$ embryos. Spina bifida occurs more frequently in *Rpl10a* $^{LOF/+}$ *Vangl2* $^{Lp/+}$ compound heterozygotes compared to *Vangl2* $^{Lp/+}$ embryos (Fisher's exact test p value = 0.3). **(e)** Frequency of looped tail phenotype in *Rps6* $^{lox/+}$, *T Cre; Rps6* $^{lox/+}$, *Vangl2* $^{Lp/+}$*; Rps6* $^{lox/+}$, and *Vangl2* $^{Lp/+}$; *T Cre; Rps6* $^{lox/+}$ embryos. The looped tail phenotype does not differ significantly between *Vangl2* $^{Lp/+}$*; Rps6* $^{lox/+}$, and *Vangl2* $^{Lp/+}$; *T Cre; Rps6* $^{lox/+}$ embryos (Fisher's exact test p value =0.3). ** = p value < 0.01. Source data are provided as a Source Data file.

Supplementary Table 1: Primers and oligos

Oligo name	Sequence	Use
Nupl1 qPCR F	catggctgcaacacttacacagca	qPCR
Nupl1 qPCR R	attgcaagccagtgccaatacctg	qPCR
Pbgd qPCR F	ggagccatgtctggtaacgg	qPCR
Pbgd qPCR R	ccacgcgaatcactctcatct	qPCR
Oct4 qPCR F	agtgagaggcaacctggaga	qPCR
Oct4 qPCR R	acactcggaccacatccttc	qPCR
Nanog qPCR F	catgagtgtggatccagcttg	qPCR
Nanog qPCR R	cctgaataagcagatccatgg	qPCR
Utf1 qPCR F	accagctgctgaccttgaac	qPCR
Utf1 qPCR R	ttgaacgtacccaagaacga	qPCR
Brachyury qPCR F	tgcttccctgagacccagtt	qPCR
Brachyury qPCR R	gatcacttctttcctttgcatcaag	qPCR
Mixl1 qPCR F	ggtaccccgacatccacttg	qPCR
Mixl1 qPCR R	taatctccggcctagccaaa	qPCR
Sox17 qPCR F	cgcacggaatttgaacagta	qPCR
Sox17 qPCR R	ggatcagggacctgtcacac	qPCR
Hhex qPCR F	cacccgacgcccttttacat	qPCR
Hhex qPCR R	gaaggctggatggatcggc	qPCR
Cdx2 qPCR F	gggctctctgagaggcaggt	qPCR
Cdx2 qPCR R	cctttgctctgcggttctg	qPCR
Hoxc6 qPCR F	acccctggatgcagcgaatgaattcg	qPCR
Hoxc6 qPCR R	gttccagggtctggtaccgcgagta	qPCR
Hoxd13 qPCR F	accagccacaggggtcccactttt	qPCR
Hoxd13 qPCR R	acgccgccgcttgtccttgtta	qPCR
Msgn1 qPCR F	cggaattacctgccacctgt	qPCR
Msgn1 qPCR R	ggtctgtgagttccccgatg	qPCR
Tbx6 qPCR F	aagtaccaaccccgcataca	qPCR
Tbx6 qPCR R	taggctgtcacggagatgaa	qPCR
Pax3 qPCR F	ctccacgctccggatagttc	qPCR
Pax3 qPCR R	atcttgtggcggatgtggtt	qPCR
Meox1 qPCR F	tctgagcgccaggtcaaag	qPCR
Meox1 qPCR R	ctgaacttggagaggctgtgg	qPCR
Foxc2 qPCR F	cctcctggtatctcaaccaca	qPCR
Foxc2 qPCR R	gagggtcgagttctcaatccc	qPCR
Sox9 qPCR F	cgtcaacggctccagcaagaacaa	qPCR
Sox9 qPCR R	gccgcttctcgctctcgttcagaagt	qPCR
Uncx4.1 qPCR F	ctatcccgacgtgttcatgc	qPCR
Uncx4.1 qPCR R	gaactcgggactcgaccag	qPCR

Twist1 qPCR F	ctgcagcaccggcaccgttt	qPCR
Twist1 qPCR R	cccaacggctggacgcacac	qPCR
Pax1 qPCR F	cgctatggagcagacgtatggcga	qPCR
Pax1 qPCR R	aatgcgcaagcggatggcgttg	qPCR
L10A-1 F	gaccctcagaaggacaaacg	Rpl10a gDNA and transcript qPCR
L10A-1 R	agaacgcacaccgagaactt	Rpl10a gDNA and transcript qPCR
L10A-2 F	gaaaacatggtggccaaagt	Rpl10a gDNA qPCR
L10A-2 R	ctaatacagacgctggggct	Rpl10a gDNA qPCR
Ptms qPCR F	ccggaaagaacggaagaaag	Rpl10a gDNA qPCR external control
Ptms qPCR R	cctctccatcctctgcagtt	Rpl10a gDNA qPCR external control
18S rRNA qPCR F	acatccaaggaaggcagcag	qPCR
18S rRNA qPCR R	cattccaattacagggcctc	qPCR
Rpl5 qPCR F	cccgaactacaactggcaat	qPCR
Rpl5 qPCR R	cattctgacccatgatgtgc	qPCR
Rps10 qPCR F	tttttaaggagggcgtgatg	qPCR
Rps10 qPCR R	atgccctcgttcgtaaggta	qPCR
Rluc F	tggagaataacttcttcgtgga	qPCR
Rluc R	ttggacgacgaacttcacc	qPCR
Mouse Actb qPCR F	gccaaccgtgaaaagatgac	qPCR
Mouse Actb qPCR R	catcacaatgcctgtggtac	qPCR
Gapdh qPCR F	acagtccatgccatcactgcc	qPCR
Gapdh qPCR R	gcctgcttcaccaccttcttg	qPCR
Vangl2 qPCR F	atctttgcatccatggctcgtg	qPCR
Vangl2 qPCR R	gccaaatatcgctccaggaag	qPCR
Fzd3 qPCR F	agatgtttggtgtcccgtgg	qPCR
Fzd3 qPCR R	cacaagtcggggatatggct	qPCR
Human Actb qPCR F	gccaaccgcgagaagatgac	qPCR
Human Actb qPCR R	catcacgatgccagtggtac	qPCR
Fgfr1 qPCR F	gagtgacttccacagccaga	qPCR
Fgfr1 qPCR R	tggagtcagcagacactgtt	qPCR
Smad4 qPCR F	ccagctctgttagccccatc	qPCR
Smad4 qPCR R	tactggcaggctgacttgtg	qPCR
Tgfbr1 qPCR F	aaccgcactgtcattcacca	qPCR
Tgfbr1 qPCR R	agcaatggtaaacctgagcca	qPCR
Dhcr24 qPCR F	tccaacacatctgcactgct	qPCR
Dhcr24 qPCR R	ggtctgagttttcggacgga	qPCR
Rac1 qPCR F	tacgcccctatcctatccg	qPCR
Rac1 qPCR R	caatcggcttgtctttgccc	qPCR
Sfrp2 qPCR F	gtttcccccaggacaacgac	qPCR
Sfrp2 qPCR R	gcaggcttcacatacctttgg	qPCR

Axin2 qPCR F	gagagtgagcggcagagc	qPCR
Axin2 qPCR R	cggctgactcgttctcct	qPCR
Universal miRNA Cloning Linker (NEB S1315S)	5′- rAppCTGTAGGCACCATCAAT–NH2-3′	Ribosome Profiling
Reverse Transcription Primer1_ATCACG	/5phos/DDDNNCGTGATNNNNTACCCTTC GCTTCACACACAAG/iSp18/GGATCC/iSp18 /TACCTGATTGATGGTGC	Ribosome Profiling
Reverse Transcription Primer2_CGATGT	/5phos/DDDNNACATCGNNNNTACCCTTC GCTTCACACACAAG/iSp18/GGATCC/iSp18 /TACCTGATTGATGGTGC	Ribosome Profiling
Reverse Transcription Primer3_TCTGAC	/5phos/DDDNNGTCAGANNNNTACCCTTC GCTTCACACACAAG/iSp18/GGATCC/iSp18 /TACCTGATTGATGGTGC	Ribosome Profiling
Reverse Transcription Primer4_GAGCTA	/5phos/DDDNNTAGCTCNNNNTACCCTTC GCTTCACACACAAG/iSp18/GGATCC/iSp18 /TACCTGATTGATGGTGC	Ribosome Profiling
Reverse Transcription Primer5_AGTCCT	/5phos/DDDNNAGGACTNNNNTACCCTTC GCTTCACACACAAG/iSp18/GGATCC/iSp18 /TACCTGATTGATGGTGC	Ribosome Profiling
Reverse Transcription Primer7_TACGGA	/5phos/DDDNNTCCGTANNNNTACCCTTC GCTTCACACACAAG/iSp18/GGATCC/iSp18 /TACCTGATTGATGGTGC	Ribosome Profiling
Reverse Transcription Primer8_CTGTAC	/5phos/DDDNNGTACAGNNNNTACCCTTC GCTTCACACACAAG/iSp18/GGATCC/iSp18 /TACCTGATTGATGGTGC	Ribosome Profiling
Reverse Transcription Primer11_TCACTG	/5phos/DDDNNCAGTGANNNNTACCCTTC GCTTCACACACAAG/iSp18/GGATCC/iSp18 /TACCTGATTGATGGTGC	Ribosome Profiling
PCR Primer P6Solexa_F	aatgatacggcgaccaccgagatctacactctttccccttgtgtgt gaagcgaagggta	Ribosome Profiling
PCR Primer P3Solexa_R	caagcagaagacggcatacgagatcggtctcggcattcctgattg atggtgcctacag	Ribosome Profiling
Sequencing Primer P6_custom_SeqPrimer	cactctttccccttgtgtgtgaagcgaagggta	Ribosome Profiling
oNTI199	auguacacggagucgacccgcaacgcga	Ribosome Profiling
oNTI265	auguacacggagucgagcucaacccgcaacgcga	Ribosome Profiling
OS57	aaaccgctcacttgcggcgcttgc	Rpl10a LOF and null mouse genotyping
OS60	agcagggagaaatccaatcc	Rpl10a LOF and null mouse genotyping
OS127	caagaaacagctacagtggctt	Rpl10a conditional and deletion mouse genotyping
OS349	cataacgataccacgatatcaaca	Rpl10a conditional and deletion mouse genotyping
FloxedS6 WT F	gcttctacttctaagtctgagtccagtc	Rps6 conditional mouse genotyping
FloxedS6 Mut F	tcctgccgagaaagtatccatcatg	Rps6 conditional mouse genotyping
FloxedS6 WT Mut2 R	ctgcagccttttcttttagcatacctg	Rps6 conditional mouse genotyping
WUSTL Cre F	gcattaccggtcgatgcaacgagtgatgag	T Cre mouse genotyping
WUSTL Cre R	gagtgaacgaacctggtcgaaatcagtgcg	T Cre mouse genotyping
Fabpi-200 F	tggacaggactggacctctgctttcctaga	T Cre mouse genotyping

Fabpi-200 R	tagagctttgccacatcacaggtcattcag	T Cre mouse genotyping
meox1creF1	ctccgcaaaacccaaatgggc	Meox1 Cre mouse genotyping
En2SArevsor	ctccaacctccgcaaactcc	Meox1 Cre mouse genotyping
oIMR9020	aagggagctgcagtggagta	Ai9 mouse genotyping
oIMR9021	ccgaaaatctgtgggaagtc	Ai9 mouse genotyping
oIMR9103	ggcattaaagcagcgtatcc	Ai9 mouse genotyping
oIMR9105	ctgttcctgtacggcatgg	Ai9 mouse genotyping
Axin2-LacZ 9391	aagctgcgtcggatacttgaga	Axin2 LacZ mouse genotyping
Axin2-LacZ 9392	agtccatcttcattccgcctagc	Axin2 LacZ mouse genotyping
Axin2-LacZ 9393	tggtaatgctgcagtggcttg	Axin2 LacZ mouse genotyping
Lp-48765 F	tggctgtcttctgcactcac	Vangl2 Lp mouse genotyping
Lp-48766 R	gcaccttcttggtgctcact	Vangl2 Lp mouse genotyping
