# Supplementary material for: A stem cell roadmap of ribosome heterogeneity reveals a function for RPL10A in mesoderm production
Source: Nat Commun. 2022 Sep 19;13:5491. doi: 10.1038/s41467-022-33263-3 (PMC9485161; doi:10.1038/s41467-022-33263-3)
Supplement: Supplementary file 2 — Description of Additional Supplementary Files [file 41467_2022_33263_MOESM2_ESM.pdf]

Description of Additional Supplementary Files

File name: Supplementary Data 1
Description: Relative quantification of each ribosomal protein from polysome, cytoplasmic extract, whole cell extract, monosome, and free subunit TMT mass spectrometry samples and relative quantification of each ribosomal protein at the mRNA level. Values are the ratios for each differentiated cell type relative to hESCs.

File name: Supplementary Data 2
Description: RNA-Seq and ribosome profiling results from E8.5 *Rpl10a* $^{LOF/LOF}$ and wild-type embryos. For each gene the log2 fold change in the RNA, log2 fold change in the ribosome protected footprints, and their respective FDRs are shown.

File name: Supplementary Data 3
Description: CAMERA Gene set enrichment analysis results using translation efficiencies measured by E8.5 *Rpl10a* $^{LOF/LOF}$ and wild-type RNA-Seq and ribosome profiling.

File name: Supplementary Movie 1
Description: Location of the 31 RPs heterogeneous in the polysome mass spectrometry data on the human 80S ribosome (PDB: 4v6x, http://doi.org/10.2210/pdb4V6X/pdb). Small and large subunit heterogeneous RPs are colored in blue and red respectively.
