# Supplementary material for: A stem cell roadmap of ribosome heterogeneity reveals a function for RPL10A in mesoderm production
Source: Nat Commun. 2022 Sep 19;13:5491. doi: 10.1038/s41467-022-33263-3 (PMC9485161; doi:10.1038/s41467-022-33263-3)
Supplement: Supplementary file 7 — Reporting Summary [file 41467_2022_33263_MOESM7_ESM.pdf]

nature portfolio

Corresponding author(s): Maria Barna

Last updated by author(s): Aug 27, 2022

Reporting Summary

Nature Portfolio wishes to improve the reproducibility of the work that we publish. This form provides structure for consistency and transparency in reporting. For further information on Nature Portfolio policies, see our Editorial Policies and the Editorial Policy Checklist.

Statistics

For all statistical analyses, confirm that the following items are present in the figure legend, table legend, main text, or Methods section.

n/a	Confirmed	
☐	☒	The exact sample size (*n*) for each experimental group/condition, given as a discrete number and unit of measurement
☐	☒	A statement on whether measurements were taken from distinct samples or whether the same sample was measured repeatedly
☐	☒	The statistical test(s) used AND whether they are one- or two-sided<br>*Only common tests should be described solely by name; describe more complex techniques in the Methods section.*
☒	☐	A description of all covariates tested
☐	☒	A description of any assumptions or corrections, such as tests of normality and adjustment for multiple comparisons
☐	☒	A full description of the statistical parameters including central tendency (e.g. means) or other basic estimates (e.g. regression coefficient) AND variation (e.g. standard deviation) or associated estimates of uncertainty (e.g. confidence intervals)
☐	☒	For null hypothesis testing, the test statistic (e.g. *F*, *t*, *r*) with confidence intervals, effect sizes, degrees of freedom and *P* value noted<br>*Give P values as exact values whenever suitable.*
☐	☒	For Bayesian analysis, information on the choice of priors and Markov chain Monte Carlo settings
☐	☒	For hierarchical and complex designs, identification of the appropriate level for tests and full reporting of outcomes
☐	☒	Estimates of effect sizes (e.g. Cohen's *d*, Pearson's *r*), indicating how they were calculated

Our web collection on statistics for biologists contains articles on many of the points above.

Software and code

Policy information about availability of computer code

Data collection	All data collection is described in the Methods section. Data collection for mass spectrometry was performed using Xcalibur (Thermo Scientific, v4.1). Western blots were detected with a ChemiDoc MP (Biorad). Sucrose density gradient data were aquired with a Density Gradient Fraction System (Brandel Cat# BR-188) and Peak Chart software (Brandel, v1.02).
Data analysis	All data analysis is described in the Methods section. Mass spectrometry data was analyzed using Proteome Discoverer 1.4 (Thermo Scientific). O-propargyl-puromycin incorporation was analyzed using FlowJo (v10.7.1). RNA-seq and ribosome profiling alignment was performed using STAR (version 2.7.6a) or bowtie2; reads processed with Skewer (v0.1.17), rsem-calculate-expression (version 1.3.3), UMI-tools (v0.5.4), FastX-toolkit (v0.0.13), cutadapt (v1.14); and further analyzed using the following packages in R (v3.6.2) in RStudio: edgeR, limma, voom, sva, locfdr. Gene set enrichment analysis was performed in R using CAMERA and visualized using Enrichment Map and Cytoscape (v3.8.2). Trachea transmission electron microscopy analysis of basal feet orientation was performed using ImageJ (v1.52a), Oriana (Kovach Computing Services), and the R package Circular. Additional data visualization and statistical analysis was performed in Excel 2013 or RStudio. Ribosome structural analysis was performed using PyMol (version 2.3.3) and PDBePISA (v1.48).

For manuscripts utilizing custom algorithms or software that are central to the research but not yet described in published literature, software must be made available to editors and reviewers. We strongly encourage code deposition in a community repository (e.g. GitHub). See the Nature Portfolio guidelines for submitting code & software for further information.

Data

Policy information about availability of data

All manuscripts must include a data availability statement. This statement should provide the following information, where applicable:

- Accession codes, unique identifiers, or web links for publicly available datasets
- A description of any restrictions on data availability
- For clinical datasets or third party data, please ensure that the statement adheres to our policy

Data availability: All processed mass spectrometry and sequencing data are available in the supplementary data files. Raw sequencing data has been deposited in the Gene Expression Omnibus under accession number GSE177520. Raw mass spectrometry data has been deposited in the ProteomeXchange with identifier PXD032904. The structure of the human ribosome utilized here was accessed from the Protein Data Bank (PDB: 4v6x, http://doi.org/10.2210/pdb4V6X/pdb). Materials may be obtained from the corresponding author upon request. Source data are provided with this paper.

Field-specific reporting

Please select the one below that is the best fit for your research. If you are not sure, read the appropriate sections before making your selection.

☒ Life sciences ☐ Behavioural & social sciences ☐ Ecological, evolutionary & environmental sciences

For a reference copy of the document with all sections, see nature.com/documents/nr-reporting-summary-flat.pdf

Life sciences study design

All studies must disclose on these points even when the disclosure is negative.

Sample size	No sample-size calculation was performed. Sample sizes for all experiments are indicated in the Methods and figure legends and all samples used for quantitative analysis are independent biological replicates. The number of samples used were determined based on standards in the field, with typically at least 3 biological replicates utilized.
Data exclusions	One out of four wild-type and one out of four homozygous mutant ribosome profiling and matched RNA-sequencing library set was excluded due to poor RNA quality, resulting in three wild-type and three homozygous mutant replicates being presented in this paper.
Replication	At least 3 independent replicates were performed for each experiment unless otherwise noted in the text. Mouse embryo experiments were performed on multiple litters from distinct parents harvested on distinct days. All attempts at replication were successful.
Randomization	For human embryonic stem cell differentiation experiments, a single cell suspension was prepared and used as a stock to aliquot across distinct dishes, and these dishes were allocated randomly for either differentiation or continued growth as embryonic stem cells (controls). For mouse embryo experiments, randomization is not relevant because all embryos from the same litter were used for the same experiment.
Blinding	Because most experiments were performed by a single individual, blinding was usually not feasible. In this study blinding is only relevant to experiments that quantify features between mouse embryos of distinct genotypes. This was achieved for quantification of global protein synthesis by OP-puromycin incorporation by having one individual prepare the samples and a second individual who was unaware of the genotypes perform flow cytometry. For all other experiments, either all steps were performed by the same individual or embryos of different genotypes were demarcated by overt phenotypic differences that made blinding impractical.

Reporting for specific materials, systems and methods

We require information from authors about some types of materials, experimental systems and methods used in many studies. Here, indicate whether each material, system or method listed is relevant to your study. If you are not sure if a list item applies to your research, read the appropriate section before selecting a response.

Materials & experimental systems

n/a	Involved in the study	
☐	☒	Antibodies
☐	☒	Eukaryotic cell lines
☒	☐	Palaeontology and archaeology
☐	☒	Animals and other organisms
☒	☐	Human research participants
☒	☐	Clinical data
☒	☐	Dual use research of concern

Methods

n/a	Involved in the study	
☒	☐	ChIP-seq
☒	☐	Flow cytometry
☒	☐	MRI-based neuroimaging

Antibodies

Antibodies used	Primary antibodies used: anti-RPL10A (Abcam ab174318, 1:1000 dilution), anti-RPL10A (Santa Cruz Biotechnology sc-100827, 1:1000 dilution), anti-RPL11 (Abcam ab79352, 1:1000 dilution), anti-RPL34 (Abcam ab129394, 1:500 dilution), anti-RPL23A (Bethyl A303-932A-M, 1:2000 dilution), anti-RPL22 (ProteinTech 25002-1-AP), anti-RPS25 (Sigma HPA031801, 1:250 dilution), anti-RPS5 (Abcam ab58345, 1:1000 dilution), anti-GAPDH (Invitrogen AM4300, 1:2000 dilution), anti-β-actin (Cell Signaling 3700S, 1:2000 dilution). Secondary antibodies used: donkey anti-mouse (GE Healthcare NA931-1ML, 1:500 dilution) or donkey anti-rabbit (GE Healthcare NA934-1ML, 1:500 dilution). For in situ hybridizations, the DIG-labeled RNA probes were detected using AP-anti-DIG (Roche 11093274910 1:2000 dilution).
Validation	Antibodies had bands of the correct molecular weight in Western blots and have been used in multiple publications. Details from manufacturers' are as follows:<br>Abcam anti-RPL10A: Rabbit monoclonal [EPR12344] to RPL10A. Suitable for: Flow Cyt (Intra), ICC, WB, IHC-P. Reacts with: Mouse, Rat, Human.<br>Santa Cruz Biotechnology anti-RPL10A: Raised against recombinant ribosomal protein l10a of human origin. Applications: ELISA, FC/FACS, IF, IHC, IHC-P, IP, WB. Reactivity: Human, House mouse, Rat.<br>anti-RPL11: Immunogen: Synthetic peptide corresponding to Human RPL11 aa 150 to the C-terminus (C terminal) conjugated to keyhole limpet haemocyanin. Suitable for: IHC-P, WB, IP. Reacts with: Human, Zebrafish. Predicted to work with: Mouse, Rat, Cow, Cat, Orangutan.<br>anti-RPL34: Immunogen: Synthetic peptide conjugated to KLH, corresponding to a region within internal sequence amino acids 36-66 of Human RPL34. Suitable for: WB, IHC-P. Reacts with: Human.<br>anti-RPL23A: Immunogen: between residue 106 and 156 of human Ribosomal Protein L23a. Tested applications: WB, IP. Species reactivity: Human. Based on 100% sequence identity, this antibody is predicted to react with Mouse, Rat and Bovine.<br>anti-RPL22: Immunogen: RPL22 fusion protein Ag21851. Tested applications: WB, IP, IF. Tested reactivity: Human. Cited reactivity: Human, Mouse.<br>anti-RPS25: Immunogen: ribosomal protein S25 recombinant protein epitope signature tag (PrEST). Techniques: WB, IF, IHC. Species reactivity: human, mouse, rat.<br>anti-RPS5: Immunogen: recombinant fragment corresponding to amino acids 106-205 of Human RPS5. Suitable for: WB, IP. Species reactivity: Human<br>anti-GAPDH: Immunogen: Purified rabbit muscle GAPDH (whole molecule). Applications: WB, IHC, ICC/IF, IP. Species reactivity: Amphibian, Dog, Chicken, Fish, Human, Mouse, Non-human primate, Rabbit, Rat.<br>anti-β-actin: Immunogen: synthetic peptide corresponding to amino-terminal residues of human β-actin. Applications: WB, IHC-P, IF, F. Species reactivity: Human, Mouse, Rat, Hamster, Monkey, Dog.<br>AP-anti-DIG: Use Anti-Digoxigenin-AP, Fab fragments for the detection of digoxigenin-labeled compounds using: cDNA array; Colony/plaque hybridization; Dot blot; ELISA; Gel shift assay; Immunohistocytochemistry; In situ hybridization; Nonradioactive DNA sequencing blot; Northern blot; RNase protection assay; Southern blot; Western blot; Fluorescent in situ hybridization; Section in situ hybridization and whole mount in situ hybridization; Electrophoretic mobility shift assay. The polyclonal antibody from sheep is specific to digoxigenin and digoxin and shows no cross-reactivity with other steroids, such as human estrogens and androgens. Nonspecific binding to RNA is not expected.

Eukaryotic cell lines

Policy information about cell lines

Cell line source(s)	H7 hESCs were originally provided by Kyle Loh's lab at Stanford University. H7 hESCs are commercially available from WiCell (catalog #WA07).
Authentication	Cell lines were not authenticated.
Mycoplasma contamination	Cell lines were not tested for mycoplasma.
Commonly misidentified lines (See ICLAC register)	No commonly misidentified cell lines were used in this study.

Animals and other organisms

Policy information about studies involving animals; ARRIVE guidelines recommended for reporting animal research

Laboratory animals	This study used the following previously published mouse lines: ROSA26::FLPe (JAX #009086), CMV-Cre (JAX #006054), Meox1 Cre, Ai9 (JAX #007909), T Cre, Rps6 lox, Axin2 lacZ (JAX #009120), and Vangl2 Lp (JAX # 000220). The Rpl10a mouse lines developed in this paper were made on C57BL/6 background. All experiments were performed on embryos at the following stages depending on the experiment: E8.5, E9.5, E10.5, E12.5, E14.5, E17.5, or E18.5 (the staging for each experiment is specified in the text). The genders were not determined.
Wild animals	The study did not involve wild animals.
Field-collected samples	The study did not involve samples collected from the field.
Ethics oversight	All mouse work was reviewed and approved by the Stanford Administrative Panel on Laboratory Animal Care (APLAC) (protocol number 27463).

Note that full information on the approval of the study protocol must also be provided in the manuscript.
